# New Monoterpenoids and Polyketides from the Deep-Sea Sediment-Derived Fungus *Aspergillus sydowii* MCCC 3A00324

**DOI:** 10.3390/md18110561

**Published:** 2020-11-17

**Authors:** Siwen Niu, Longhe Yang, Tingting Chen, Bihong Hong, Shengxiang Pei, Zongze Shao, Gaiyun Zhang

**Affiliations:** 1 Key Laboratory of Marine Genetic Resources, Third Institute of Oceanography, Ministry of Natural Resources, 184 Daxue Road, Xiamen 361005, China; peishengxiang@stu.xmu.edu.cn (S.P.); shaozongze@tio.org.cn (Z.S.); 2 Technology Innovation Center for Exploitation of Marine Biological Resources, Third Institute of Oceanography, Ministry of Natural Resources, Xiamen 361005, China; longheyang@tio.org.cn (L.Y.); chentingting@tio.org.cn (T.C.)

**Keywords:** polyketides, monoterpenoids, deep-sea fungus, *Aspergillus sydowii*, ECD calculation

## Abstract

Chemical study of the secondary metabolites of a deep-sea-derived fungus *Aspergillus sydowii* MCCC 3A00324 led to the isolation of eleven compounds (**1**–**11**), including one novel (**1**) and one new (**2**) osmane-related monoterpenoids and two undescribed polyketides (**3** and **4**). The structures of the metabolites were determined by comprehensive analyses of the NMR and HRESIMS spectra, in association with quantum chemical calculations of the ^13^C NMR, ECD, and specific rotation data for the configurational assignment. Compound **1** possessed a novel monoterpenoid skeleton, biogenetically probably derived from the osmane-type monoperpenoid after the cyclopentane ring cleavage and oxidation reactions. Additionally, compound **3** was the first example of the *α*-pyrone derivatives bearing two phenyl units at C-3 and C-5, respectively. The anti-inflammatory activities of **1**–**11** were tested. As a result, compound **6** showed potent inhibitory nitric oxide production in lipopolysaccharide (LPS)-activated BV-2 microglia cells with an inhibition rate of 94.4% at the concentration of 10 µM. In addition, a plausible biosynthetic pathway for **1** and **2** was also proposed.

## 1. Introduction

**Figure 1:**
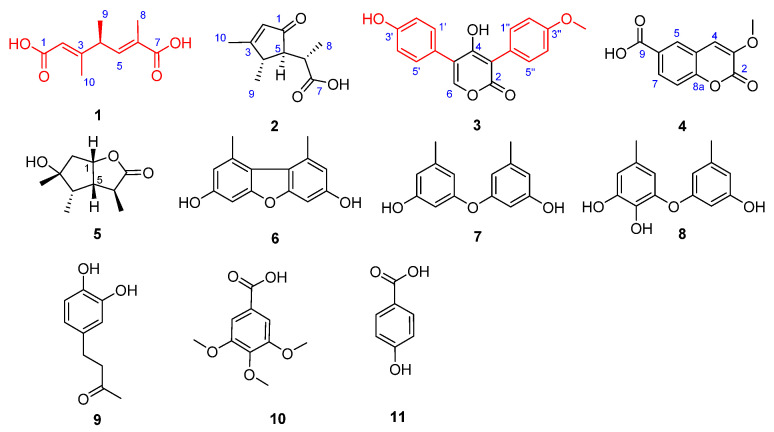
Chemical structures of **1**–**11** from *Aspergillus sydowii* MCCC 3A00324.

Deep-sea-derived fungi inhabiting extreme sea environments have proven to be a new source for a wide array of structurally intriguing and biologically active secondary metabolites [1,2,3]. As of December 2019, about 700 new secondary metabolites were reported from deep-sea-derived fungi, such as terpenoids, polyketides, alkaloids, and steroids. Some of the metabolites featured a broad range of biological activities, for example, cytotoxicity [4,5] as well as antiviral [6,7], antibacterial [8,9], anti-inflammatory [10], and antiallergic effects [11]. In our continuing efforts to discover new or bioactive secondary metabolites from deep-sea-derived fungi [12,13,14,15,16], the fungus *Aspergillus sydowii* MCCC 3A00324, isolated from the South Atlantic Ocean deep-sea sediment (2246 m), attracted our attention due to the abundantly metabolic profile obtained by HPLC and TLC analysis (Figure S1, Supplementary Materials). A literature retrieval discovered that 52 out of 59 new compounds were isolated from the marine-derived *Aspergillus sydowii* fungus. The bisabolane-type sesquiterpenoids [17,18,19,20,21,22,23,24], xanthones [18,23,25], diphenyl ethers [20,26,27,28], and diketopiperazine alkaloids [29,30,31] were common secondary metabolites of the *A. sydowii*, some of which exhibited a wide range of pharmacological activities. To date, only three undescribed polyketides, namely asperentin B [32], 2,3,5-trimethyl-6-(3-oxobutan-2-yl)-4H-pyran-4-one, and (2*R*)-2,3-dihydro-7-hydroxy-6,8-dimethyl-2-[(*E*)-propl-enyl] chromen-4-one [33], have been reported from the deep-sea-derived *A. sydowii*. On the basis of the seldomly carried out chemical investigation and the abundantly metabolic profile of the *A. sydowii* MCCC 3A00324, a chemical study of the MCCC 3A00324 fungus was carried out. In our previous research, 17 undescribed and 10 known sesquiterpenoids were obtained from the fermented cultures of the fungus [34]. In our continuous research on this fungus, eleven compounds (**1**–**11**) (Figure 1), including one novel (**1**) and one new (**2**) monoterpenoids as well as two new polyketides (**3** and **4**) were discovered. Compounds **1** and **2** were the first representatives of the osmane-related monoterpenoids discovered from the fungi, while **3** was the first example of the *α*-pyrone derivatives with two phenyl units at C-3 and C-5, respectively. As terpenoids usually possess anti-inflammatory activities [35,36,37], all compounds were subsequently evaluated for their anti-inflammatory effects. As a result, compound **6** exhibited strong inhibitory nitric oxide production in lipopolysaccharide (LPS)-activated BV-2 microglia cells with a 94.4% inhibition rate at 10 µM. Herein, the isolation, structure elucidation, and anti-inflammatory activities of these metabolites as well as the biosynthetic pathway of **1** and **2** are reported.

## 2. Results and Discussion

**Figure 2:**
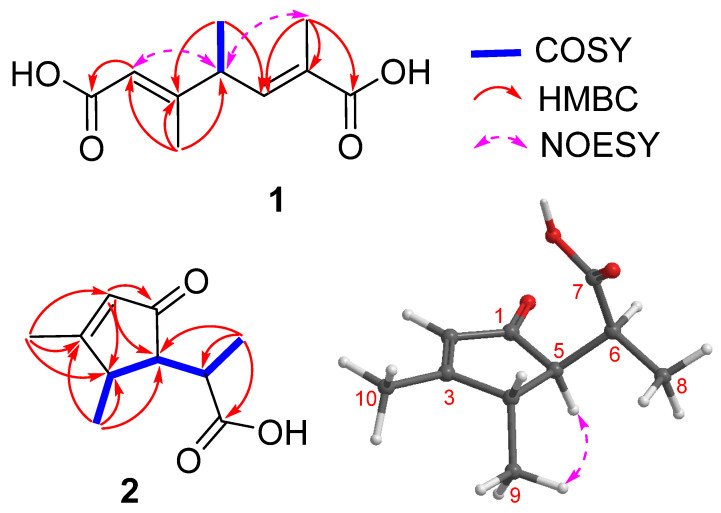
Key COSY, HMBC, and NOESY correlations of **1** and **2**.

Aspermonoterpenoid A (**1**), colorless oil, has the molecular formula of C_10_H_14_O_4_ as determined by the ^13^C NMR data and the HRESIMS spectrum (*m*/*z* 221.0784, [M + Na]^+^), indicating four degrees of unsaturation. The ^1^H NMR spectrum showed two olefinic protons (*δ*_H_ 5.77, 6.66), a methine (*δ*_H_ 3.36), and three methyls (*δ*_H_ 1.24, 1.88, 2.14) (Table 1), while the ^13^C NMR and HSQC spectra exhibited four sp^2^ carbons (*δ*_C_ 117.0, 130.2, 144.5, 162.0) for two double bonds, two ester carbonyl carbons (*δ*_C_ 170.4, 171.5), one methine (*δ*_C_ 43.8), and three methyls (*δ*_C_ 12.8, 17.0, 19.0) (Table 1). As all degrees of unsaturation were accounted for by two carbonyl carbons and two double bonds, an acyclic structure was required in **1**. The COSY data of H-4 (*δ*_H_ 3.36)/H_3_-9 (*δ*_H_ 1.24) and the HMBC cross-peaks from H_3_-8 (*δ*_H_ 1.88) to C-5 (*δ*_C_ 144.5)/C-6 (*δ*_C_ 130.2)/C-7 (*δ*_C_ 171.5), H_3_-9 to C-3 (*δ*_C_ 162.0)/C-4 (*δ*_C_ 43.8)/C-5, H_3_-10 (*δ*_H_ 2.14) to C-2 (*δ*_C_ 117.0)/C-3/C-4, and from H-2 (*δ*_H_ 5.77) to C-1 (*δ*_C_ 170.4)/C-4/C-10 (*δ*_C_ 17.0) established the planar structure of **1** as 2,4,5-trimethylhepta-2,5-dienedioic acid (Figure 2). The *E* geometries at Δ^2^ and Δ^5^ were resolved on the basis of the NOESY cross-peaks from H-2 to H-4 and from H_3_-8 to H-4 (Figure 2), which were further corroborated by the chemical shifts of Me-8 (*δ*_C_ 12.8) and Me-10 (*δ*_C_ 17.0). The sole chiral center of C-4 in **1** was resolved by the calculation of the specific rotation data using Gaussian 09 [38]. The specific rotation data of (4S*)-**1** was calculated at the B3LYP/6-311+G(2d,p) level with the CPCM model in methanol, which has the same sign ([*α*${]}_{D}$ +138) as that of the experimental data ([*α*${]}_{D}^{25}$ +54), revealing the 4*S* configuration. Therefore, the structure of **1** was determined to be (2*E*,5*E*,4*S*)-2,4,5-trimethylhepta-2,5-dienedioic acid, which was named aspermonoterpenoid A. Noteworthily, **1** possessed a novel chained monoperpenoid skeleton, biogenetically probably derived from the osmane-type monoperpenoid after the cyclopentane ring cleavage and oxidation reactions [39,40].

**Figure 3:**
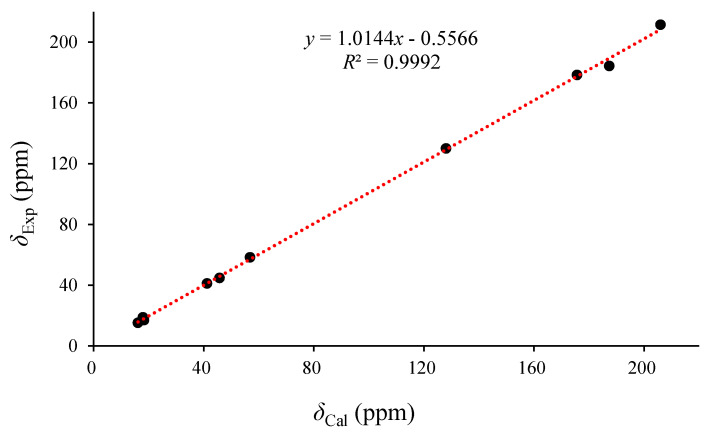
Linear regression analysis of the experimental ^13^C NMR data of **2** and calculated ^13^C NMR chemical shift data of **2a**.

**Figure 4:**
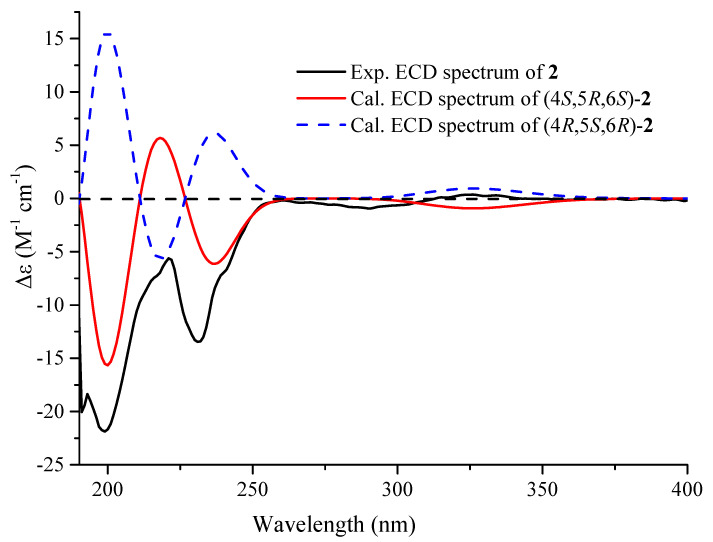
Experimental (Exp.) and calculated (Cal.) ECD spectra of **2** in methanol at the B3LYP/6-311+G(2d,p) level.

The molecular formula of aspermonoterpenoid B (**2**) was established to be C_10_H_14_O_3_ on the basis of the HRESIMS spectrum at the sodium adduct ion peak at *m*/*z* 205.0845 (calcd for C_10_H_14_O_3_Na, 205.0841), requiring four degrees of hydrogen deficiency. The ^1^H NMR spectrum showed one olefinic proton (*δ*_H_ 5.89), three methines (*δ*_H_ 2.21, 2.76, 2.94), and three methyls (*δ*_H_ 1.28, 1.29, 2.13). Analyses of the ^13^C NMR spectrum, with the aid of the HSQC spectrum, revealed ten carbon signals attributable to two olefinic carbons (*δ*_C_ 130.1, 184.4) for a double bond, two carbonyl carbons for a keto (*δ*_C_ 211.6) and a carboxylic (*δ*_C_ 178.5) functionalities, three methines (*δ*_C_ 41.2, 44.8, 58.4), and three methyls (*δ*_C_ 15.3, 17.1, 18.9), which accounted for three degrees of unsaturation. The remaining one double bond equivalent indicated that a monocyclic skeleton was required in **2**. The COSY cross-peaks of H-4 (*δ*_H_ 2.76)/H-5 (*δ*_H_ 2.21)/H-6 (*δ*_H_ 2.94) and the HMBC correlations from H_3_-8 (*δ*_H_ 1.29) to C-5 (*δ*_C_ 58.4)/C-6 (*δ*_C_ 41.2)/C-7 (*δ*_C_ 178.5), H_3_-9 (*δ*_H_ 1.28) to C-3 (*δ*_C_ 184.4)/C-4 (*δ*_C_ 44.8)/C-5, H_3_-10 (*δ*_H_ 2.13) to C-2 (*δ*_C_ 130.1)/C-3/C-4, and from H-2 (*δ*_H_ 5.89) to C-1 (*δ*_C_ 211.6)/C-4/C-5 deduced the structure of **2** to be 3,4-dimethyl-5-isopropanic acidated cyclopentenone (Figure 2). The relative configuration of C-4 and C-5 in cyclopentenone ring moiety was determined as 4S* and 5R* on the basis of the NOESY correlation from H_3_-9 to H-5, in addition to the very weak interaction between H-4 and H-5 (Figure 2). Additionally, the relative configuration of C-6 was unreliable to be deduced by the NOESY data because of its location at the soft side chain. In order to assign the relative configuration, the two possible epimers (4S*,5R*,6S*)-**2** (**2a**) and (4S*,5R*,6R*)-**2** (**2b**) were subjected to the ^13^C NMR chemical shift theoretical calculation at the mPW1PW91/6-311+G(2d,p) level in methanol using Gaussian 09. As shown in Figure 3, the calculated ^13^C NMR data of **2a** were nearly identical to those of the experimental NMR data, as corroborated by the correlation coefficient (*R*^2^) and root mean square error (RMSE) of **2a** (*R*^2^ = 0.9992, RMSE = 2.4436) and **2b** (*R*^2^ = 0.9988, RMSE = 2.7912), suggesting the 4S*, 5R*, and 6S* configurations. The absolute configuration of **2** was resolved on the basis of the ECD calculation, which was carried out at the B3LYP/6-311+G(2d,p) level in MeOH using the optimized geometries at the B3LYP/6-31+G(d,p) level in gas phase after conformational searches via the OPLS3 force field using Maestro 10.2. The ECD spectrum of **2a** was calculated by the TDDFT method, which was in good agreement with the experimental ECD curve, indicating the 4*S,* 5*R*, and 6*S* configurations (Figure 4). Interestingly, **2** was the first representative of the osmane-type monoterpenoids discovered from the fungi.

**Figure 5:**
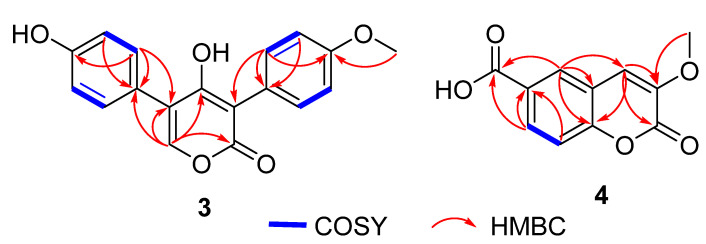
Selected COSY and HMBC correlations of compounds **3** and **4**.

Compound **3** was isolated as a colorless oil, and its molecular formula was assigned to be C_18_H_14_O_5_ on the basis of the HRESIMS spectrum at the sodium adduct ion peak at *m*/*z* 333.0741, implying 12 indices of hydrogen deficiency. The ^1^H NMR spectrum exhibited four aromatic protons with dual intensity at *δ*_H_ 6.85 (d, *J* = 8.5 Hz, 2H), 7.01 (d, *J* = 8.6 Hz, 2H), 7.29 (d, *J* = 8.5 Hz, 2H), and 7.33 (d, *J* = 8.6 Hz, 2H), indicating the presence of two para-substituted benzene rings. Moreover, a methoxy (*δ*_H_ 3.85) and one olefinic (*δ*_H_ 7.59) protons were also observed (Table 2). The ^13^C NMR spectrum showed a total of 18 carbon resonance signals, including twelve aromatic carbons (*δ*_C_ 115.1 × 2, 116.3 × 2, 123.5, 124.6, 131.9 × 2, 133.2 × 2, 158.9, and 160.9) for two benzene units, four olefinic carbons (*δ*_C_ 107.1, 120.4, 149.5, and 167.1) for two double bonds, a carbonyl carbon (*δ*_C_ 166.2), and one methoxy group (*δ*_C_ 55.7) (Table 2). The COSY data of H-1″ (*δ*_H_ 7.33) and H-2″ (*δ*_H_ 7.01) and the HMBC cross-peaks from H-1″/H-5″ to C-3″ (*δ*_C_ 160.9) and C-3 (*δ*_C_ 107.1), H-2″/H-4″ to C-3″ and C-6″ (*δ*_C_ 124.6), and from the methoxy protons (*δ*_H_ 3.85) to C-3″ deduced the *p*-methoxyphenyl moiety (ring C) to be tethered at C-3 (Figure 5). Additional COSY correlation between H-1′ (*δ*_H_ 7.29)/H-2′ (*δ*_H_ 6.85) and the HMBC correlations from H-1′/H-5′ to C-3′ (*δ*_C_ 158.9) and C-5 (*δ*_C_ 120.4) and from H-2′/H-4′ to C-3′ and C-6′ (*δ*_C_ 123.5) in association with the chemical shift of C-3′ established the *p*-hydroxyphenyl unit (ring A) to be resided at C-5. The remaining five olefinic carbons (*δ*_C_ 107.1, 120.4, 149.5, 166.2, and 167.1) constructed an *α*-pyrone moiety with a hydroxy at C-4 position, according to the HMBC cross-peaks from H-6 (*δ*_H_ 7.59) to C-2 (*δ*_C_ 166.2), C-4 (*δ*_C_ 167.2), C-5 (*δ*_C_ 120.4), and C-6′, as well as their chemical shifts and the molecular formula. Therefore, the structure of **3** was elucidated to be 4-hydroxy-5-(4-hydroxyphenyl)-3-(4-methoxyphenyl)-2*H*-pyran-2-one, which was named asperphenylpyrone. It is of note that **3** was the first example of the *α*-pyrone derivatives bearing two phenyl units at C-3 and C-5, respectively.

Compound **4** has the molecular formula of C_11_H_8_O_5_ as determined by the positive HRESIMS spectrum (*m*/*z* 243.0268, [M + Na]^+^) and the ^13^C NMR data, indicating eight degrees of unsaturation. The ^1^H NMR spectrum showed three aromatic protons (*δ*_H_ 7.45 (d, *J* = 8.6 Hz, H-8), 7.95 (dd, *J* = 8.6, 1.8 Hz, H-7), and 8.23 (d, *J* = 1.8 Hz, H-5)) for a 1,2,4-trisubstituted benzene ring, a singlet olefinic proton (*δ*_H_ 7.49), and one methoxy (*δ*_H_ 3.86), whereas the ^13^C NMR spectrum exhibited 11 carbon signals, including six aromatic carbons (*δ*_C_ 116.5, 120.4, 127.8, 128.9, 129.5, and 152.2) for a benzene ring, two olefinic carbons (*δ*_C_ 113.4, 144.8) for a double bond, two ester carbonyl carbons (156.6, 166.9), and a methoxy group (*δ*_C_ 56.8). The aforementioned NMR data were very similar to those of the known coumarin derivative [41], with the exception of the presence of an additional aromatic methine (*δ*_H_ 7.95, *δ*_C_ 129.5) in **4** instead of an oxygenated nonprotonated sp^2^ carbon. The additional olefinic proton was appointed at C-7 (*δ*_C_ 129.5) on the basis of the COSY cross-peaks from H-7 (*δ*_H_ 7.95) to H-8 (*δ*_H_ 7.45) and H-5 (*δ*_H_ 8.23) as well as the HMBC correlations from H-5 to C-4 (*δ*_C_ 113.4)/C-7/C-9 (*δ*_C_ 166.9)/C-8a (*δ*_C_ 152.2) and from H-7 to C-5 (*δ*_C_ 128.9), C-9, and C-8a (Figure 5). Thus, the structure of **4** was established as a 7-deoxylated derivative of the above known compound, which was given the trivial name of aspercoumarine acid.

In addition, seven known compounds were established to be pestalotiolactone A (**5**) [42,43], 3,7-dihydroxy-1,9-dimethyldibenzofuran (**6**) [44], diorcinol (**7**) [45], cordyol C (**8**) [46], 4-(3′,4′-dihydroxyphenyl)-2-butanone (**9**) [47], 3,4,5-trimethoxybenzoic acid (**10**) [48], and 4-hydroxybenzoic acid (**11**) [49] on the basis of comparison of their NMR and specific rotation data with those reported in the literature.

**Scheme 1:**
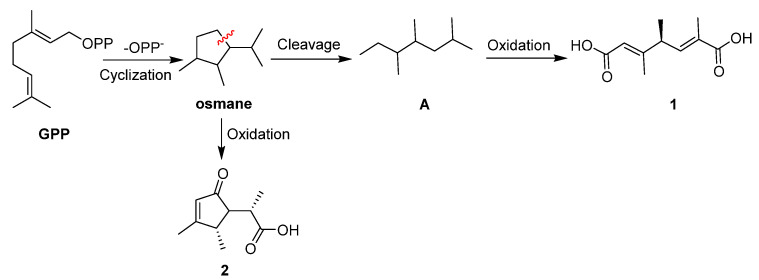
Hypothetical biogenetic pathways for compounds **1** and **2**.

Compound **1** was a novel chained monoterpenoid. The plausible biosynthetic pathway for **1** and **2** was proposed (Scheme 1). Starting from the GPP, hydrosis, oxygenation, and cyclization reaction occurrence constructed the monocyclic ring monoterpenoid osmane. The osmane might undergo carbon–carbon bond cleavage to form a key intermediate **A**, which was further oxygenated to yield **1**. Additionally, the osmane might also undergo oxygenation to form **2**.

As terpenoids usually possess the anti-inflammatory activities, all isolated metabolites were evaluated for their inhibitory effects against NO secretion in LPS-activated BV-2 microglia cells. As a result, none of them showed obvious cytotoxic activities against BV-2 microglia cells at the concentration of 20 µM by the CCK-8 Kit, and all compounds exhibited dose-dependent inhibitory effects against NO production induced by the LPS at the concentrations of 20 and 10 µM, respectively (Table 3). Interestingly, compound **6** (10 µM) showed potent anti-inflammatory activities with the inhibition rate of 94.4%.

## 3. Materials and Methods

### 3.1. General Experimental Procedures

UV spectra were recorded using a UV8000 UV/Vis spectrophotometer (Shanghai Metash Instruments Inc., Shanghai, China). Optical rotation data were measured on the basis of the Rudolph Autopol IV automatic polarimeter (Rudolph Reaearch Analytical, Newburgh, NY, USA). ECD spectra were measured using a Chirascan spectrometer (Applied Photophysics inc., Leatherhead, Surrey, UK). HRESIMS data were measured using a Xevo G2 Q-TOF mass spectrometer (Waters, Milford, MA, USA). The ^1^H, ^13^C, HSQC, COSY, HMBC, and NOESY spectra were measured on the basis of the Bruker Avance 400 FT NMR spectrometer (Bruker Company, Fällanden, Switzerland) with tetramethylsilicane (TMS) as an internal standard. Semi-preparative HPLC was performed using an Alltech LS class pump with a model 201 variable wavelength UV/Vis detector, and a YMC packed ODS-A (250 × 10 mm, 5 μm) column (YMC Co., Ltd. Kyoto, Japan) was used for the purification. Column chromatography (CC) was carried out using a Sephadex LH-20 (Amersham Biosciences, San Francisco, CA, USA), ODS-A-HG (YMC Co., Ltd. Kyoto, Japan), and silica gel (Qingdao Marine Chemistry Co., Ltd., Qingdao, China). The TLC analyses were carried out with the precoated silica gel plates by heating after spraying with vanillin sulfuric acid chromogenic reagent (Xilong Scientific Co., Ltd., Shantou, China).

### 3.2. Fungal Material and Identifiation

The fungus was isolated from the deep-sea sediment at the depth of 2246 m sampling from the South Atlantic Ocean (W13.6639°, S14.2592°), and it was identified to be *Aspergillus sydowii* on the basis of the morphology and the internal transcribed spacer (ITS) region of the rDNA sequence. The ITS gene sequence was deposited in GenBank and assigned the accession no. MN918102. The fungus was preserved at the Marine Culture Collection of China (MCCC), and assigned the accession no. MCCC 3A00324. Therefore, the producing fungus was named *Aspergillus sydowii* MCCC 3A00324.

### 3.3. Fermentation, Extraction, and Isolation

The fungus was cultivated in a potato dextrose agar (PDA) plate under 25 °C for four days, and then the fresh mycelia and spores were inoculated into 500 mL Erlenmeyer flasks (×2), each containing 100 mL potato dextrose broth (PDB) medium and followed by cultivation in a rotary shaker under 25 °C at 200 rpm for four days. The seed cultures were subsequently inoculated to 30 Erlenmeyer flasks (1 L) (each containing 80 g rice and 120 mL sea water) after autoclaving at 121 °C for 22 min. The fermentation was performed under static conditions at 25 °C for 26 days.

The fermented material was fragmented using a stick and extracted successively with EtOAc three times, and then evaporated under reduced pressure to get an EtOAc extract (16.2 g). The extract was subjected to the vacuum liquid chromatography column on silica gel eluting with a gradient of CH_2_Cl_2_ and MeOH (1:0 to 0:1) to furnish fractions A and B. The fraction B (8.5 g) was subsequently separated by CC on ODS with MeOH/H_2_O elution (30–100%) to obtain fourteen fractions (Fr.1–Fr.14). Fraction Fr.3 (147 mg) was separated by CC on silica gel eluting with petroleum ether (PE)/EtOAc (10:1) to obtain two subfractions. The former was further purified by semi-preparative HPLC with the mobile phase of MeOH/H_2_O (3:7) to yield **2** (1.8 mg) and **5** (21.0 mg), whereas the latter was separated by semi-preparative HPLC (CH_3_CN/H_2_O, 1:9) to get **11** (0.5 mg). Fraction Fr.5 (333 mg) was chromatographed by CC on silica gel eluting with CH_2_Cl_2_/MeOH (30:1) to furnish two subfractions, whereas the former was further separately purified by semi-preparative HPLC using CH_3_CN/H_2_O (19:81) elution to obtain **10** (4.0 mg), and the latter using MeOH/H_2_O (7:18) elution to yield **4** (3.0 mg). Fraction Fr.7 (489 mg) was subjected to silica gel CC with CH_2_Cl_2_ and MeOH gradient elution (1:0→0:1) to yield two subfractions (Fr.7-1 and Fr.7-2). Subfraction Fr.7-1 was separated by semi-preparative HPLC purification (CH_3_CN/H_2_O, 23:77) to get **1** (1.4 mg) and **3** (3.8 mg). Subfraction Fr.7-2 was purified by semi-preparative HPLC (CH_3_CN/H_2_O, 37:63) to yield **9** (1.2 mg). Fraction Fr.11 (146 mg) was separated by silica gel CC eluting with CH_2_Cl_2_/acetone gradient from 1:0 to 0:1 to furnish two subfractions, whereas the former was further separated using CC on a Sephadex LH-20 (MeOH) and semi-preparative HPLC (CH_3_CN/H_2_O, 3:7) to obtain **7** (18.5 mg) and **8** (3.0 mg), respectively. Fraction Fr.13 (545 mg) was subjected to CC on silica gel eluting with PE/EtOAc (1:0→0:1) to get four subfractions (Fr.13-1–Fr.13-4). Subfraction Fr.13-1 was subjected to semi-preparative HPLC with the mobile phase of CH_3_CN/H_2_O (39:61) to yield **6** (1.0 mg).

Aspermonoterpenoid A (**1**): colorless oil; [${\alpha ]}_{D}^{25}$ +54 (*c* 0.06, MeOH); UV (MeOH) *λ*_max_ (log *ε*) 223 (1.59) nm; ^1^H and ^13^C NMR data, see Table 1; HRESIMS *m*/*z* 221.0784 [M + Na]^+^ (calcd. for C_10_H_14_O_4_Na, 221.0790).

Aspermonoterpenoid B (**2**): colorless oil; [*α*${]}_{D}^{25}$ −38 (*c* 0.07, MeOH); UV (MeOH) *λ*_max_ (log *ε*) 229 (0.96) nm; ECD (MeOH) *λ*_max_ (Δ*ε*) 199 (−21.88), 222 (−5.77), 231 (−13.46), 260 (−0.15) nm; ^1^H and ^13^C NMR data, see Table 1; HRESIMS *m*/*z* 205.0845 [M + Na]^+^ (calcd. for C_10_H_14_O_3_Na, 205.0841).

Asperphenylpyrone (**3**): colorless oil; UV (MeOH) *λ*_max_ (log *ε*) 204 (1.67), 248 (0.85), 315 (0.28) nm; ^1^H and ^13^C NMR data, see Table 2; HRESIMS *m*/*z* 333.0741 [M + Na]^+^ (calcd. for C_18_H_14_O_5_Na, 333.0739).

Aspercoumarine acid (**4**): white powder; UV (MeOH) *λ*_max_ (log *ε*) 204 (0.38), 296 (0.15) nm; ^1^H and ^13^C NMR data, see Table 2; HRESIMS *m*/*z* 243.0268 [M + Na]^+^ (calcd. for C_11_H_8_O_5_Na, 243.0269).

### 3.4. BV-2 Cell Culture and Treatment

The BV-2 microglia cells were cultured in DMEM medium containing 10% fetal bovine serum and antibiotics (100 units/mL of penicillin and 100 g/mL of streptomycin) and maintained in a humidified 5% CO_2_ incubator (Beijing Luxi Technology Co., Ltd., Beijing, China) at 37 °C. For the experiment, cells were seeded into 24-well plates (2 × 10^4^ cells/well) overnight. Next day, cells were incubated with fresh culture medium containing indicated concentration of the tested compounds for half an hour and following LPS treatment (1 μg/mL). Cells were treated vehicle (DMSO, 0.1%) as control.

### 3.5. Nitrite Quantification

The concentration of nitrite in culture medium was determined using a Griess Reagent Kit (Thermo Fisher, Shanghai, China). Briefly, 75 μL of cell culture supernatants were reacted with an equal volume of Griess Reagent Kit for 30 min at room temperature, and absorbance of diazonium was obtained at a wavelength of 560 nm. Nitrite production by vehicle stimulation was designated as 100% inhibition compared to LPS stimulation for the experiment.

### 3.6. Computational Details

#### 3.6.1. ^13^C NMR Calculation of 2

Conformational searches were carried out using the Maestro 10.2 program (Schrödinger Inc., NY, USA) at the OPLS3 molecular mechanics force field within an energy window of 3.0 kcal/mol. The results exhibited 8 conformers for (4S*,5R*,6S*)-**2** and (4S*,5R*,6R*)-**2**, respectively. The conformers were further optimized at the B3LYP/6-31+G(d,p) level in gas phase using Gaussian 09 (Gaussian, Inc., Wallingford, CT, USA) [38]. The conformers with a Boltzmann population over 1% were selected for the NMR calculations. The NMR data were calculated by the GIAO method at the mPW1PW91/6-311+G(2d,p) level with the IEFPCM model in methanol. Finally, the calculated NMR data were averaged according to the Boltzmann distribution for each conformer and then fitted to the experimental values by linear regression.

#### 3.6.2. ECD Calculation of 2

The eight conformers of (4S*,5R*,6S*)-**2** were optimized by density functional theory (DFT) calculations at the B3LYP/6-31+G(d,p) level in gas phase using Gaussian 09. The energies, oscillator strengths, and rotational strengths of the first 60 electronic excitations were calculated by the TDDFT method at the B3LYP/6-311+G(2d,p) level in methanol. The ECD spectrum was simulated using SpecDis (version1.70, Berlin, Germany) by applying the Gaussian band shapes with sigma = 0.3 eV. Finally, the calculated ECD data were weighted and then summed up each stable conformer on the basis of the Boltzmann population.

## 4. Conclusions

In summary, four new compounds, including one novel (**1**) and one new (**2**) monoterpenoids and two new polyketides (**3** and **4**), were obtained from the EtOAc extract of the deep-sea-derived fungus *Aspergillus sydowii* MCCC 3A00324, together with seven known compounds (**5**–**11**). The structures of metabolites were determined by comprehensive analyses of the NMR and HRESIMS spectra, in association with quantum chemical calculations of the ECD, ^13^C NMR, and specific rotation data for their configurational assignment. Compound **1** possessed a novel monoterpenoid skeleton, biogenetically probably derived from the osmane-type monoperpenoid after the cyclopentane ring cleavage and oxidation reactions. Additionally, **2** was the first osmane-type monoterpenoid representative discovered from the fungi, while **3** was the first example of the *α*-pyrone derivatives bearing two phenyl units at C-3 and C-5, respectively, indicating that the deep-sea-derived fungi are a unique source of the structurally novel compounds. Compound **6** exhibited significant inhibitory effects against NO secretion in LPS-activated BV-2 microglia cells (94.4% inhibition rate, 10 µM), suggesting the potential application for the anti-inflammatory agents.

## Figures and Tables

**Table 1:** ^1^H (400 MHz) and ^13^C (100 MHz) NMR spectroscopic data of **1** and **2** in CD_3_OD.

No.	1		2	
	*δ*_C_	*δ*_H_	*δ*_C_	*δ*_H_
1	170.4, C		211.6, C	
2	117.0, CH	5.77, s	130.1, CH	5.89, brs
3	162.0, C		184.4, C	
4	43.8, CH	3.36, m	44.8, CH	2.76, m
5	144.5, CH	6.66, d (9.0)	58.4, CH	2.21, dd (4.9, 2.5)
6	130.2, C		41.2, CH	2.94, m
7	171.5, C		178.5, C	
8	12.8, CH_3_	1.88, d (0.6)	15.3, CH_3_	1.29, d (7.2)
9	19.0, CH_3_	1.24, d (6.8)	18.9, CH_3_	1.28, d (7.1)
10	17.0, CH_3_	2.14, s	17.1, CH_3_	2.13, s

**Table 2:** ^1^H (400 MHz) and ^13^C (100 MHz) NMR spectroscopic data of **3** and **4**.

No.	3 *^a^*		No.	4 *^b^*	
	*δ*_C_	*δ*_H_		*δ*_C_	*δ*_H_
2	166.2 *^c^*, C		2	156.6, C	
3	107.1, CH		3	144.8, C	
4	167.1 *^c^*, C		4	113.4, CH	7.49, s
5	120.4, C		4a	120.4, C	
6	149.5, CH	7.59, s	5	128.9, CH	8.23, d (1.8)
1′	131.9, CH	7.29, d (8.5)	6	127.8, C	
2′	116.3, CH	6.85, d (8.5)	7	129.5, CH	7.95, dd (8.6, 1.8)
3′	158.9, C		8	116.5, CH	7.45, d (8.6)
4′	116.3, CH	6.85, d (8.5)	8a	152.2, C	
5′	131.9, CH	7.29, d (8.5)	9	166.9, C	
6′	123.5, C		OMe	56.8, CH_3_	3.86, s
1″	133.2, CH	7.33, d (8.6)			
2″	115.1, CH	7.01, d (8.6)			
3″	160.9, C				
4″	115.1, CH	7.01, d (8.6)			
5″	133.2, CH	7.33, d (8.6)			
6″	124.6, C				
OMe	55.7, CH_3_	3.85, s			

*^a^* Measured in CD_3_OD. *^b^* Measured in DMSO-*d*_6_. *^c^* Assignments in column could be interchanged.

**Table 3:** Inhibitory effects of **1**–**11** against NO production in LPS-activated BV-2 microglia cells and their cytotoxicities against BV-2 microglia cells.

Compounds	Anti-NO (%)		Cell Viability Inhibition (%)	
	20 µM	10 µM	20 µM	10 µM
**1**	33.5 ± 1.5	10.2 ± 2.0	0.7 ± 0.1	0.3 ± 1.2
**2**	34.0 ± 1.4	22.7 ± 1.4	4.6 ± 2.6	−1.1 ± 0.4
**3**	22.7 ± 1.5	13.2 ± 1.3	4.1 ± 7.6	3.3 ± 3.1
**4**	28.3 ± 0.7	18.0 ± 2.0	3.5 ± 3.7	2.01 ± 1.4
**5**	39.1 ± 1.6	25.1 ± 0.8	10.0 ± 0.2	3.2 ± 1.7
**6**	101.4 ± 2.4	94.4 ± 0.0	−1.6 ± 5.1	−0.8 ± 3.6
**7**	55.0 ± 1.4	35.4 ± 2.4	4.1 ± 3.8	−1.5 ± 3.5
**8**	30.7 ± 0.8	18.0 ± 0.8	1.8 ± 4.7	−0.1 ± 8.2
**9**	39.3 ± 0.7	30.4 ± 1.9	2.3 ± 0.1	−1.1 ± 3.7
**10**	44.8 ± 0.7	33.0 ± 0.7	0.4 ± 1.3	0.2 ± 1.8
**11**	42.7 ± 1.3	30.8 ± 2.6	1.9 ± 2.4	−0.5 ± 3.1

## Supplementary Materials

The following are available online at https://www.mdpi.com/1660-3397/18/11/561/s1, Figure S1: The HPLC and TLC metabolic profile of the study fungus, Figures S1-1–S4-7: HRESIMS, ^1^H, ^13^C, HSQC, COSY, and HMBC spectra of new compounds **1**–**4**, and NOESY spectra of **1** and **2**.
