# Supplementary material for: New Monoterpenoids and Polyketides from the Deep-Sea Sediment-Derived Fungus Aspergillus sydowii MCCC 3A00324
Source: Mar Drugs. 2020 Nov 17;18(11):561. doi: 10.3390/md18110561 (PMC7696626; doi:10.3390/md18110561)
Supplement: Supplementary file 1 [file marinedrugs-18-00561-s001.pdf]

Supporting Information

New Monoterpenoids and Polyketides from the Deep-Sea Sediment-Derived Fungus *Aspergillus sydowii* MCCC 3A00324

Siwen Niu[a,b,†,*], Longhe Yang[b,†], Tingting Chen[b], Bihong Hong[b,*], Shengxiang Pei[a], Zongze Shao[a], Gaiyun Zhang[a,*]

[a] *Key Laboratory of Marine Genetic Resources, Third Institute of Oceanography, Ministry of Natural Resources, Xiamen 361005, People's Republic of China*

[b] *Technology Innovation Center for Exploitation of Marine Biological Resources, Third Institute of Oceanography, Ministry of Natural Resources, Xiamen 361005, People's Republic of China*

∗**Corresponding authors:** Siwen Niu, Bihong Hong, and Gaiyun Zhang

E-mail addresses: niusiwen@tio.org.cn (S. Niu), bhhong@tio.org.cn (B. Hong), and zhgyun@tio.org.cn (G. Zhang)

Author contributions

[†] S. Niu and L. Yang contributed equally to this work.

Contents | Pages

Figure S1. HPLC and TLC profiles of the extract from the deep-sea-derived *Aspergillus sydowii* MCCC 3A00324. S3

Figure S1-1. HRESIMS spectrum of **1**. S4

Figure S1-2. ^{1}H NMR spectrum of **1** in CD_3OD (400 MHz). S5

Figure S1-3. ^{13}C NMR spectrum of **1** in CD_3OD (100 MHz). S6

Figure S1-4. HSQC spectrum of **1** in CD_3OD. S7

Figure S1-5. COSY spectrum of **1** in CD_3OD. S8

Figure S1-6. HMBC spectrum of **1** in CD_3OD. S9

Figure S1-7. NOESY spectrum of **1** in CD_3OD. S10

Figure S2-1. HRESIMS spectrum of **2**. S11

Figure S2-2. ^{1}H NMR spectrum of **2** in CD_3OD (400 MHz). S12

Figure S2-3. ^{13}C NMR spectrum of **2** in CD_3OD (100 MHz). S13

Figure S2-4. HSQC spectrum of **2** in CD_3OD. S14

Figure S2-5. COSY spectrum of **2** in CD_3OD. S15

Figure S2-6. HMBC spectrum of **2** in CD_3OD. S16

Figure S2-7. NOESY spectrum of **2** in CD_3OD. S17

Figure S3-1. HRESIMS spectrum of **3**. S18

Figure S3-2. ^{1}H NMR spectrum of **3** in CD_3OD (400 MHz). S19

Figure S3-3. ^{13}C NMR spectrum of **3** in CD_3OD (100 MHz). S20

Figure S3-4. HSQC spectrum of **3** in CD_3OD. S21

Figure S3-5. COSY spectrum of **3** in CD_3OD. S22

Figure S3-6. HMBC spectrum of **3** in CD_3OD. S23

Figure S4-1. HRESIMS spectrum of **4**. S24

Figure S4-2. ^{1}H NMR spectrum of **4** in DMSO-d_6 (400 MHz). S25

Figure S4-3. ^{13}C NMR spectrum of **4** in DMSO-d_6 (100 MHz). S26

Figure S4-4. HSQC spectrum of **4** in DMSO-d_6. S27

Figure S4-5. COSY spectrum of **4** in DMSO-d_6. S28

Figure S4-6. HMBC spectrum of **4** in DMSO-d_6. S29

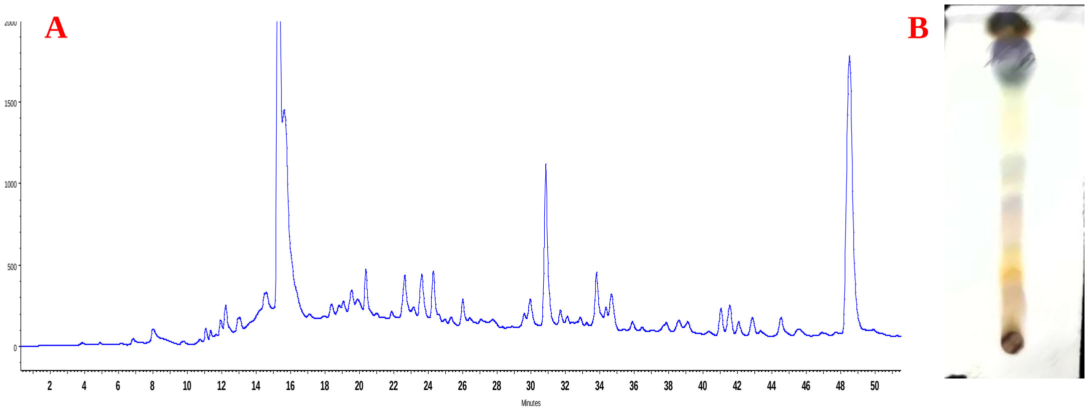

Figure S1. HPLC profile of the EtOAc extracts from the *Aspergillus sydowii* 3A00324 detected by UV absorption at 254 nm (Chromatographic conditions: MeOH/H_2O, 5%–100% 0–35 min, 100%–100% 35–45 min, 100%–5% 45–46 min, 5% 46–52 min) (**A**). TLC profile of the EtOAc extracts of the above fungus analyzed by vanillin sulfuric acid chromogenic reagent after heating (Mobile phase, CH_2Cl_2:MeOH = 10:1) (**B**).

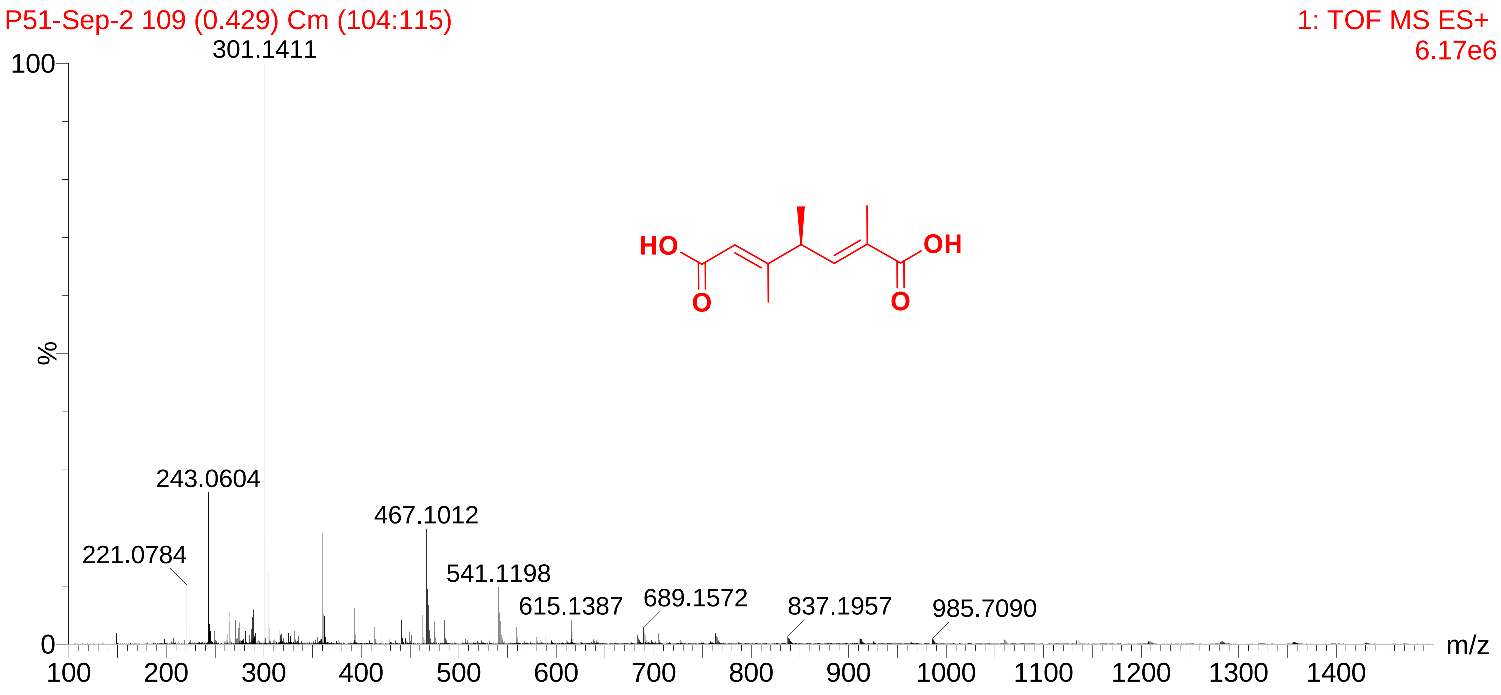

Figure S1-1. HRESIMS spectrum of **1**.

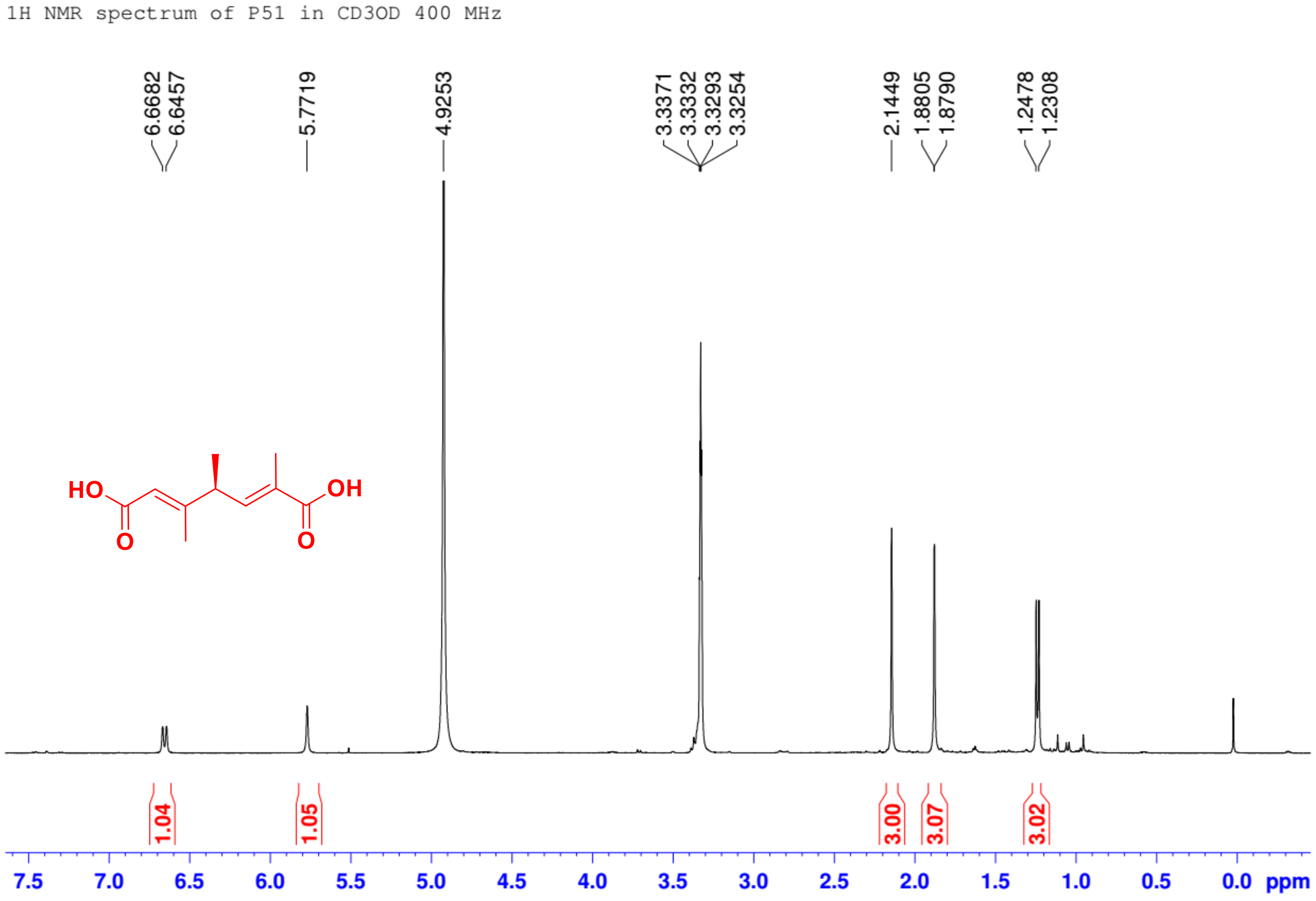

Figure S1-2. ^{1}H NMR spectrum of **1** in CD_3OD (400 MHz).

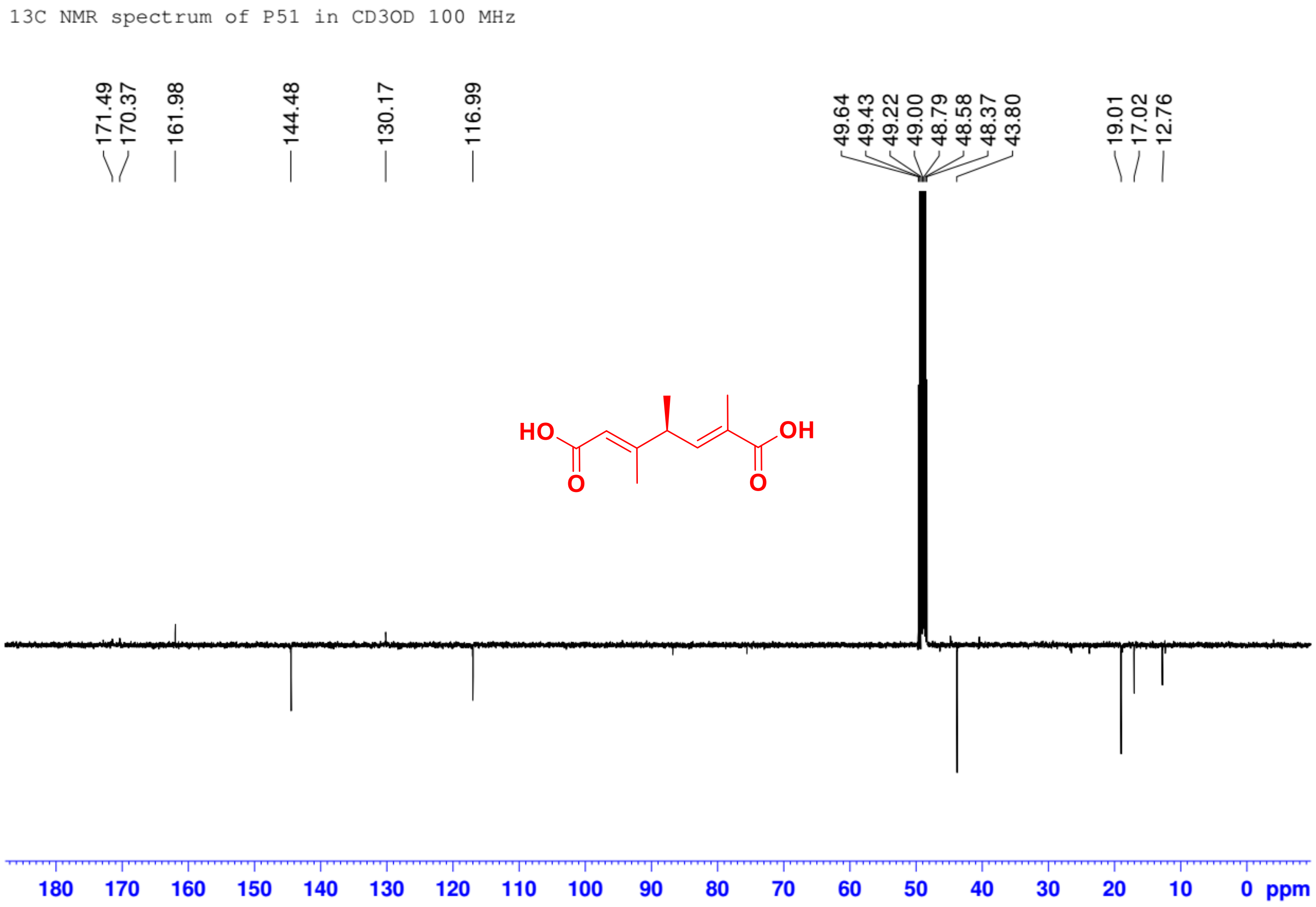

Figure S1-3. ^{13}C NMR spectrum of **1** in CD_3OD (100 MHz).

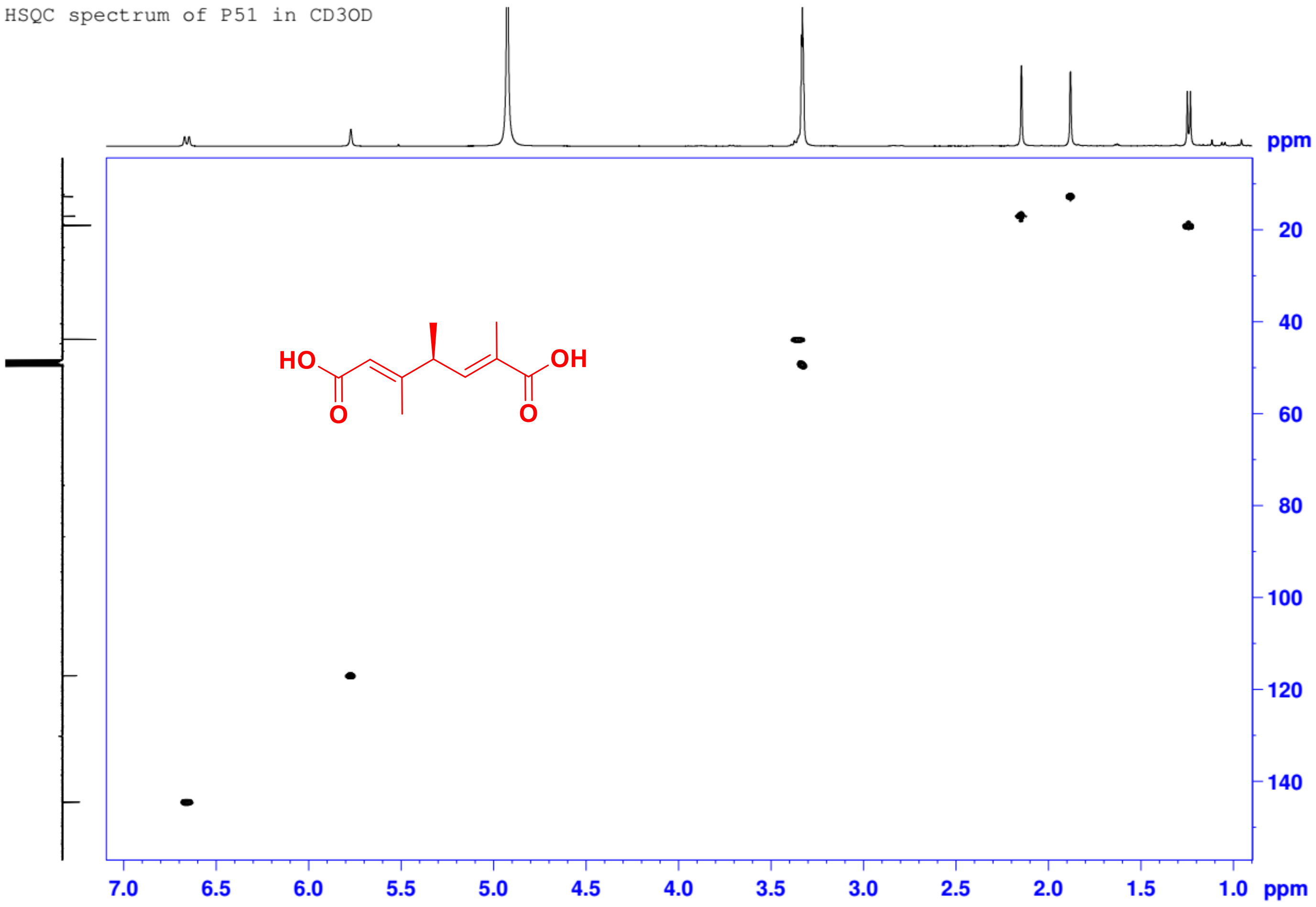

Figure S1-4. HSQC spectrum of **1** in CD_3OD.

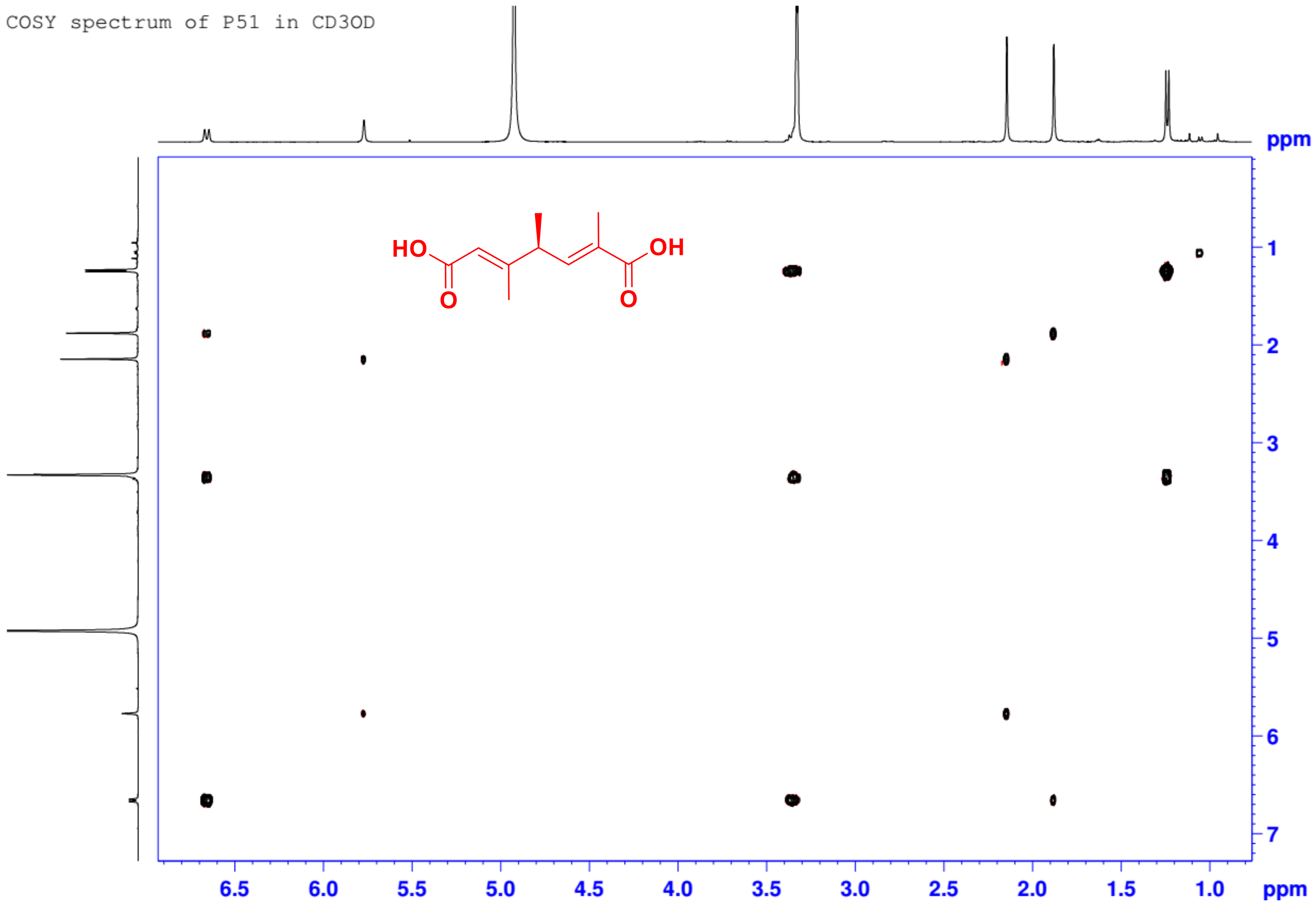

Figure S1-5. COSY spectrum of **1** in CD_3OD.

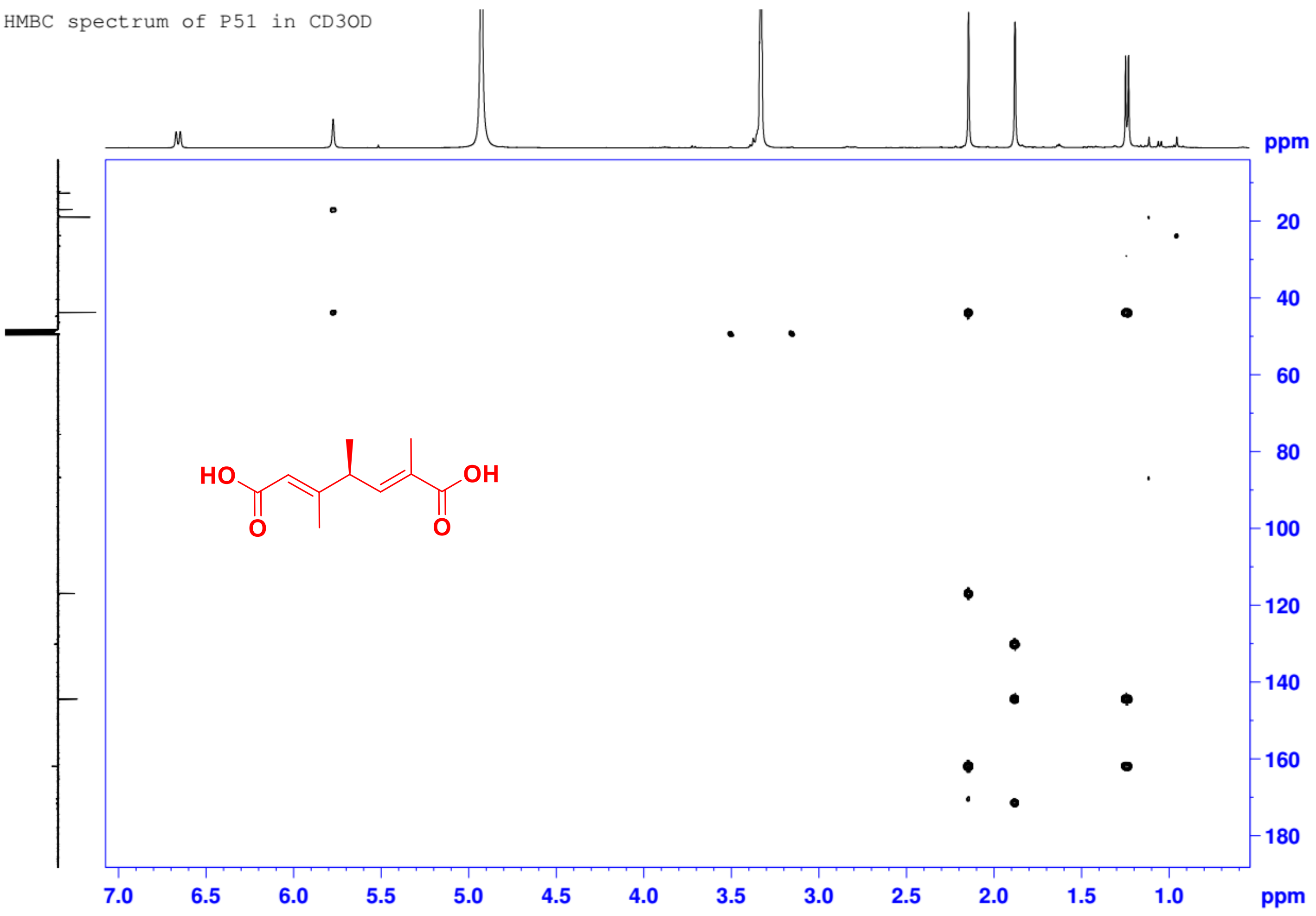

Figure S1-6. HMBC spectrum of **1** in CD_3OD.

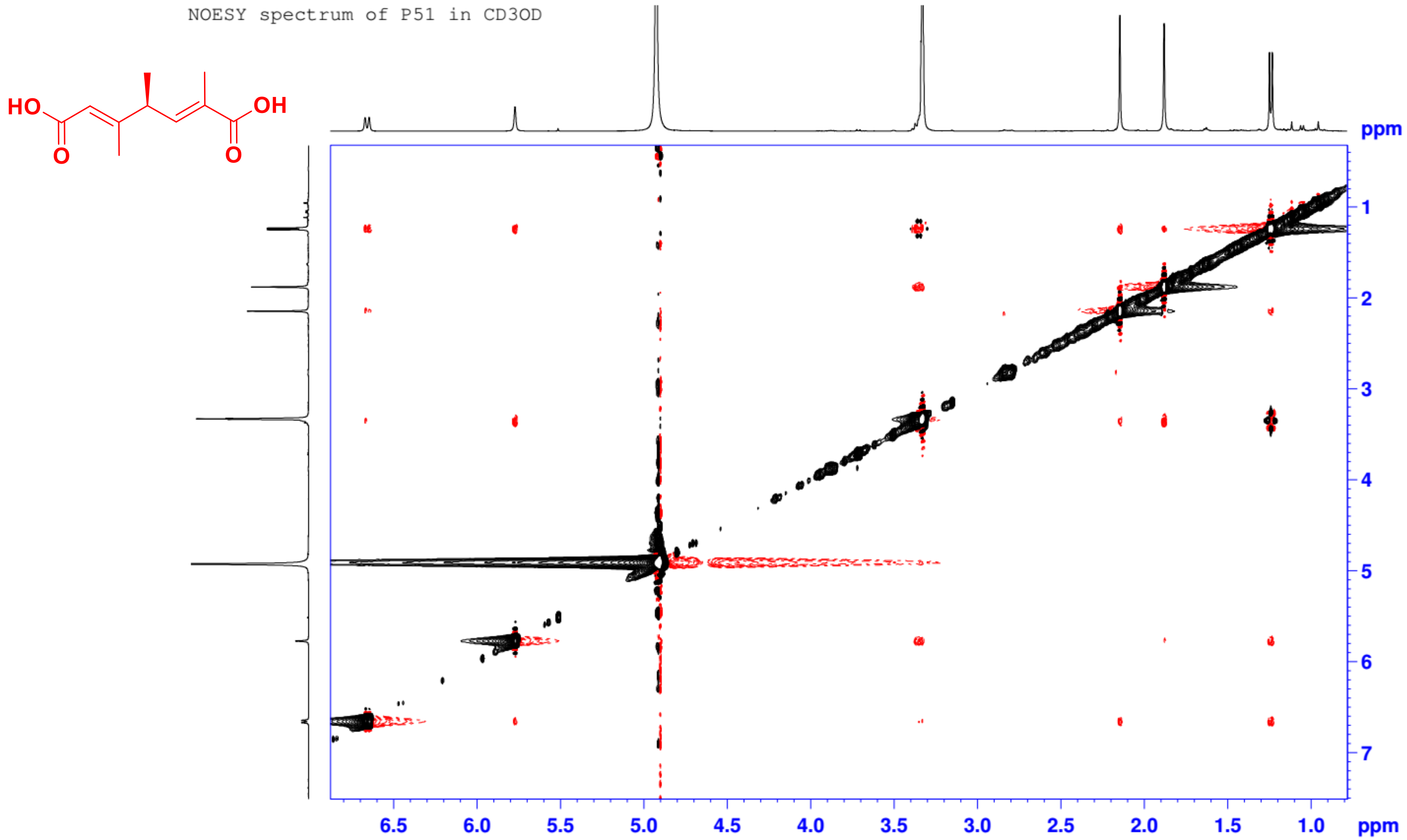

Figure S1-7. NOESY spectrum of **1** in CD_3OD.

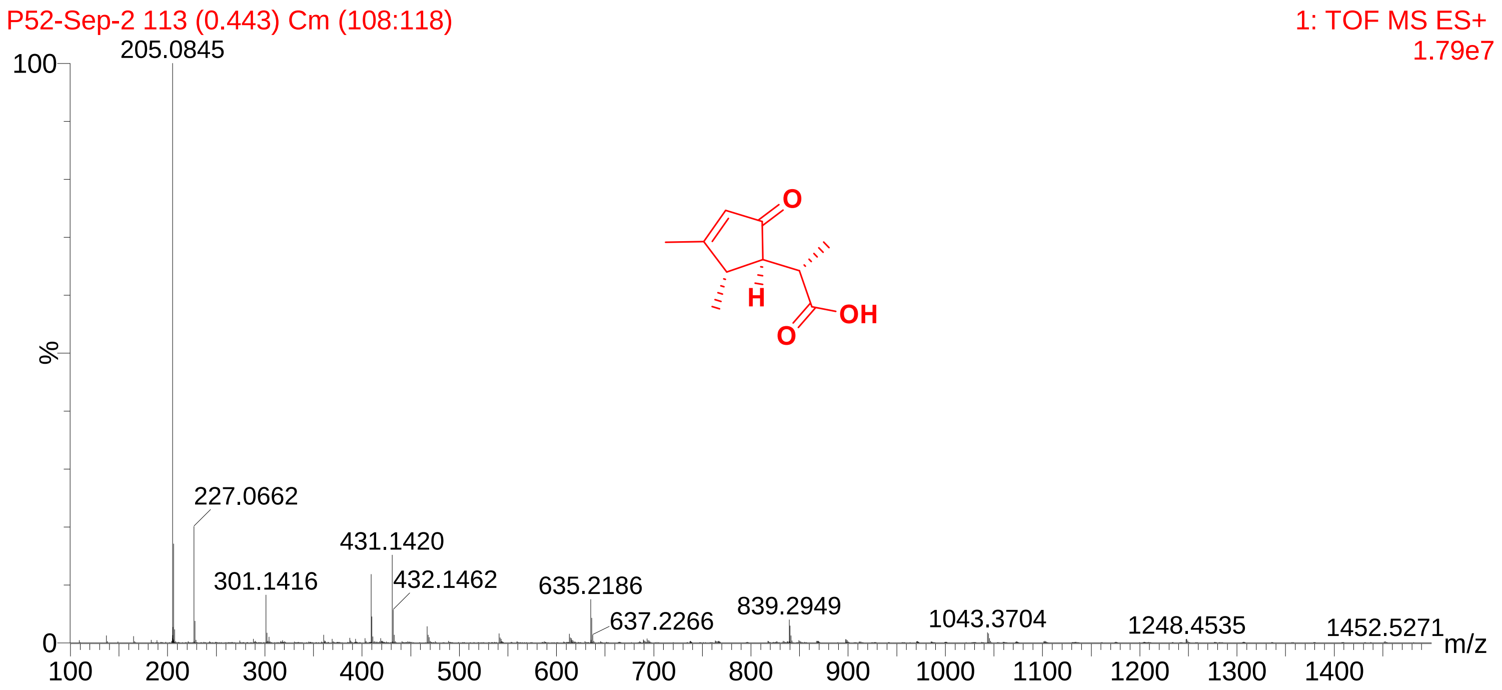

Figure S2-1. HRESIMS spectrum of **2**.

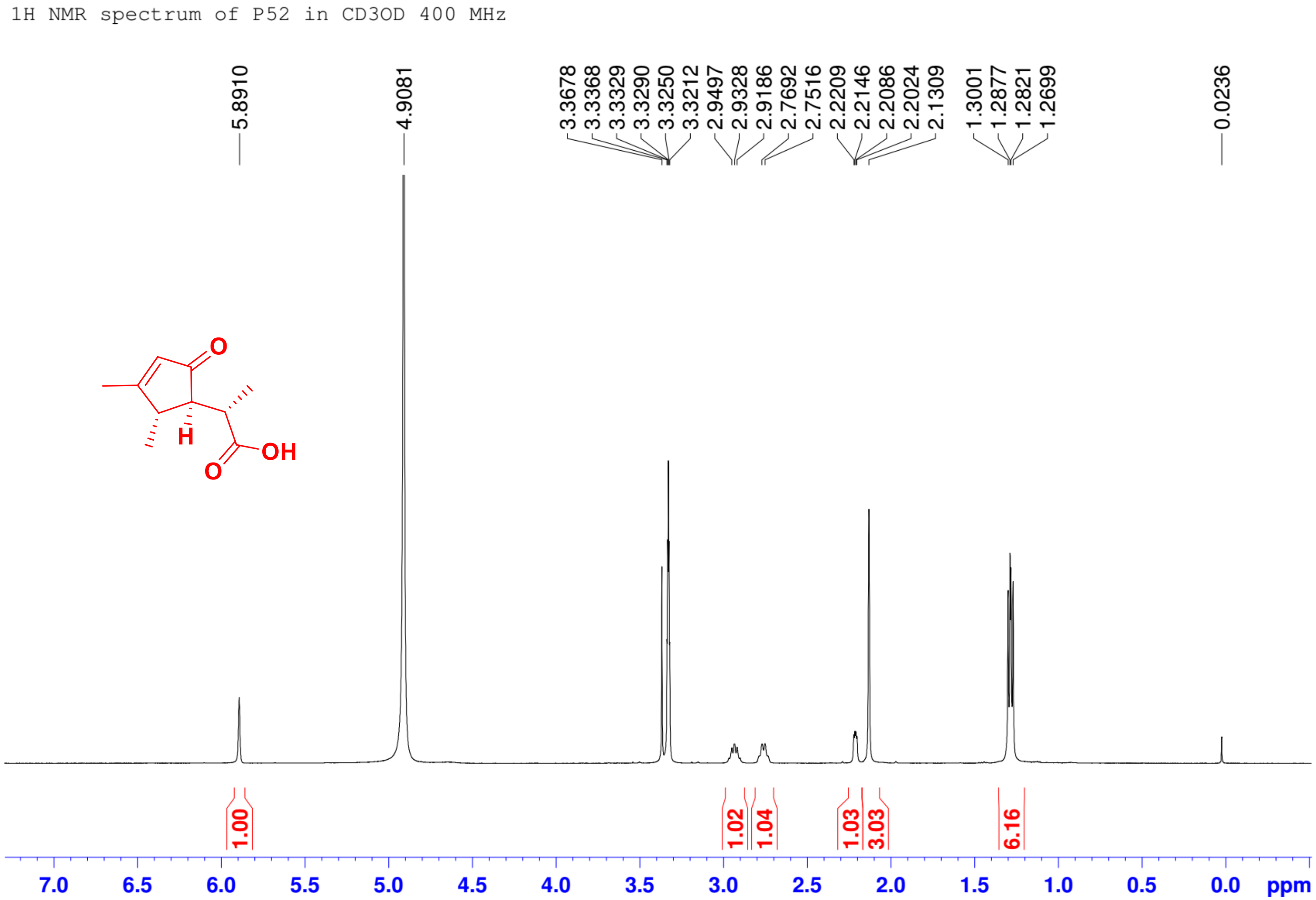

Figure S2-2. ^{1}H NMR spectrum of **2** in CD_3OD (400 MHz).

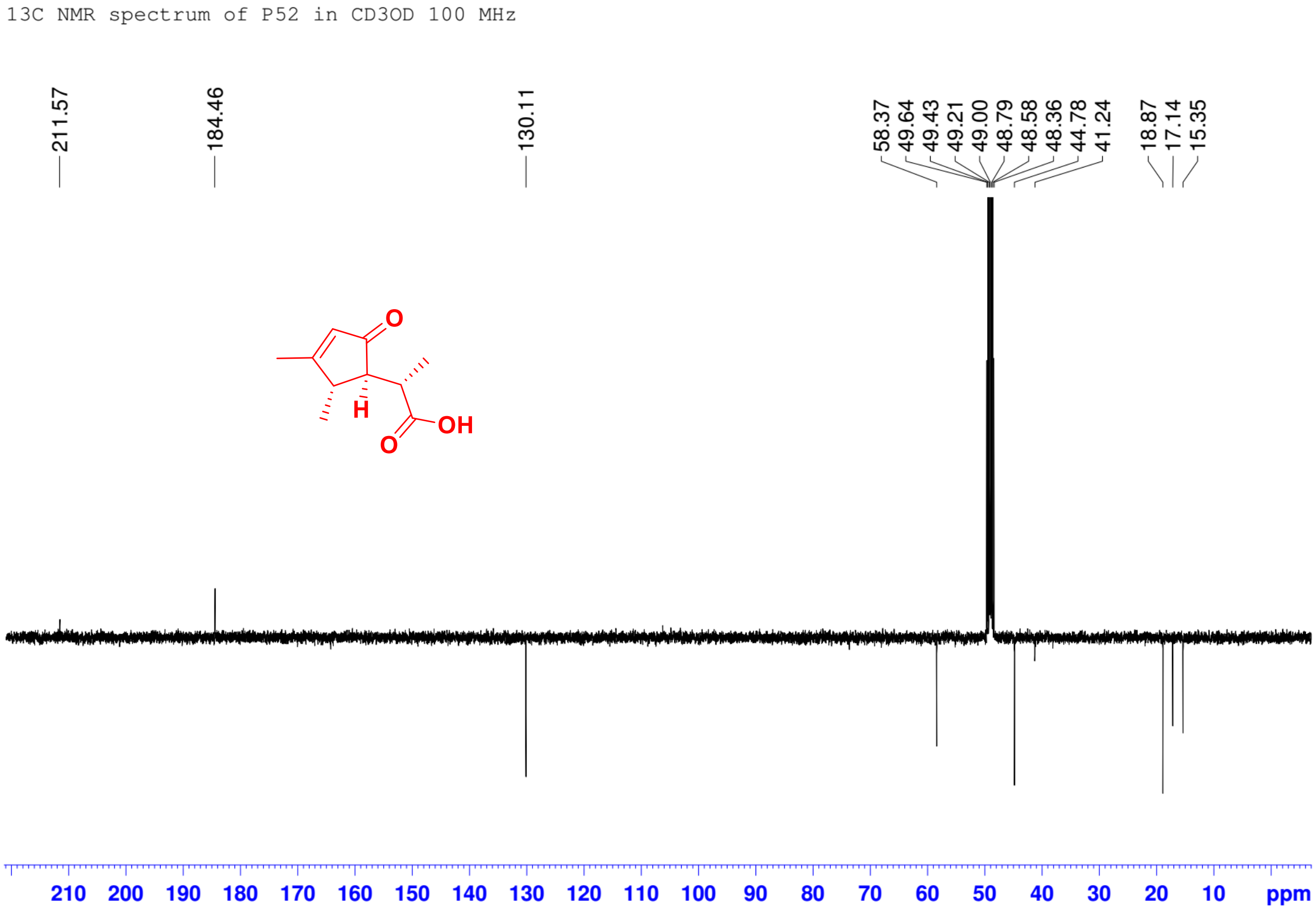

Figure S2-3. ^{13}C NMR spectrum of **2** in CD_3OD (100 MHz).

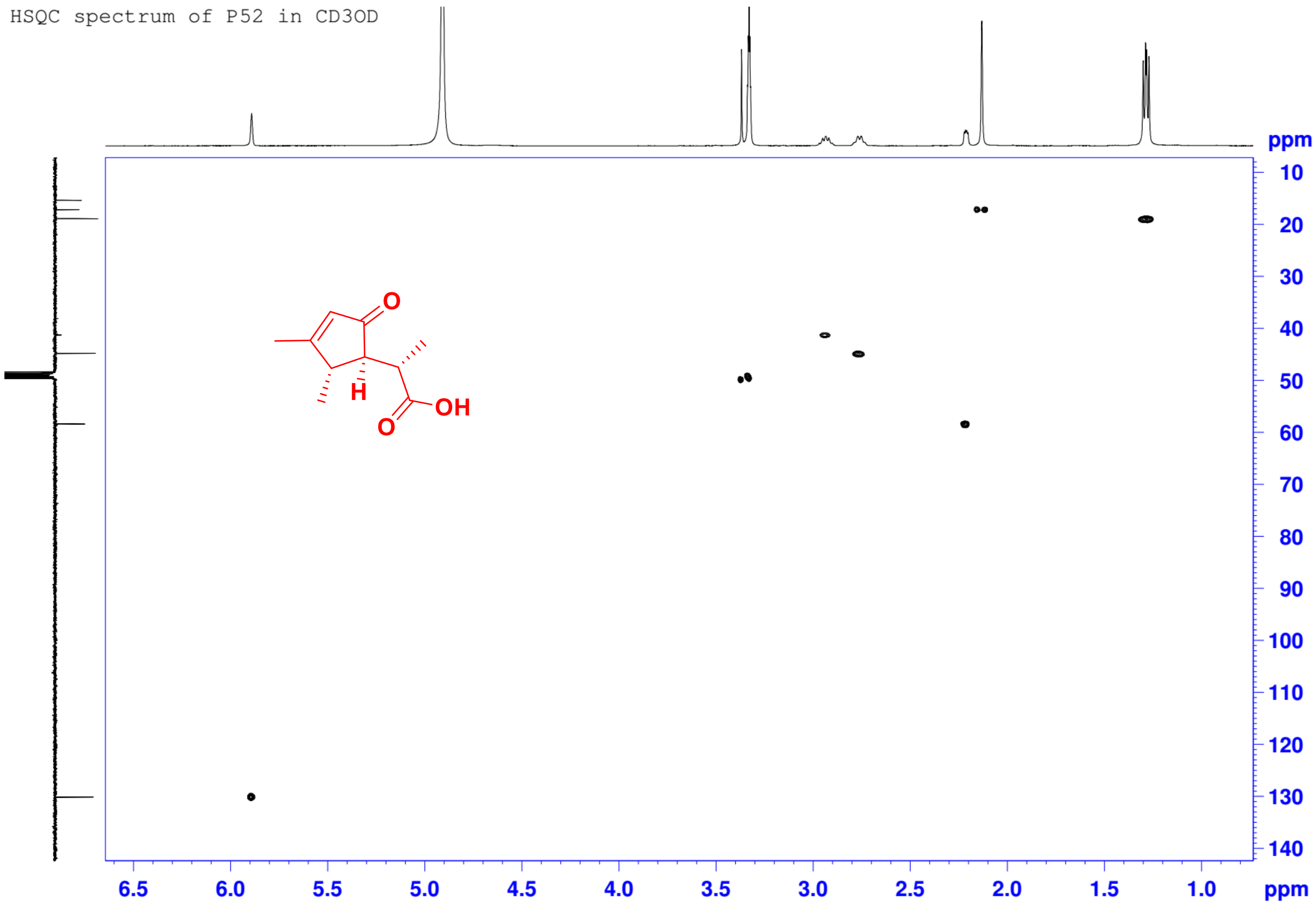

Figure S2-4. HSQC spectrum of **2** in CD_3OD.

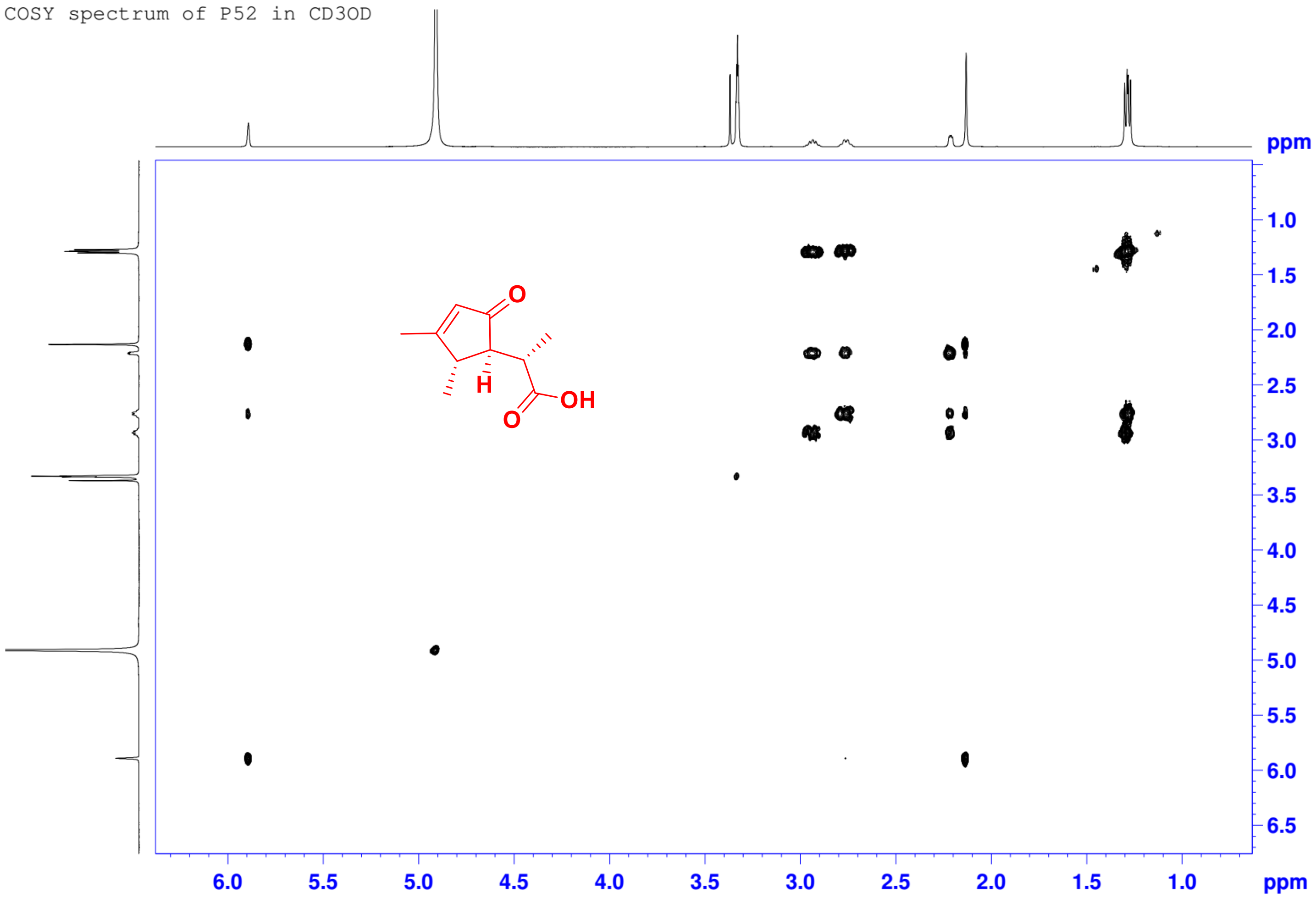

Figure S2-5. COSY spectrum of **2** in CD_3OD.

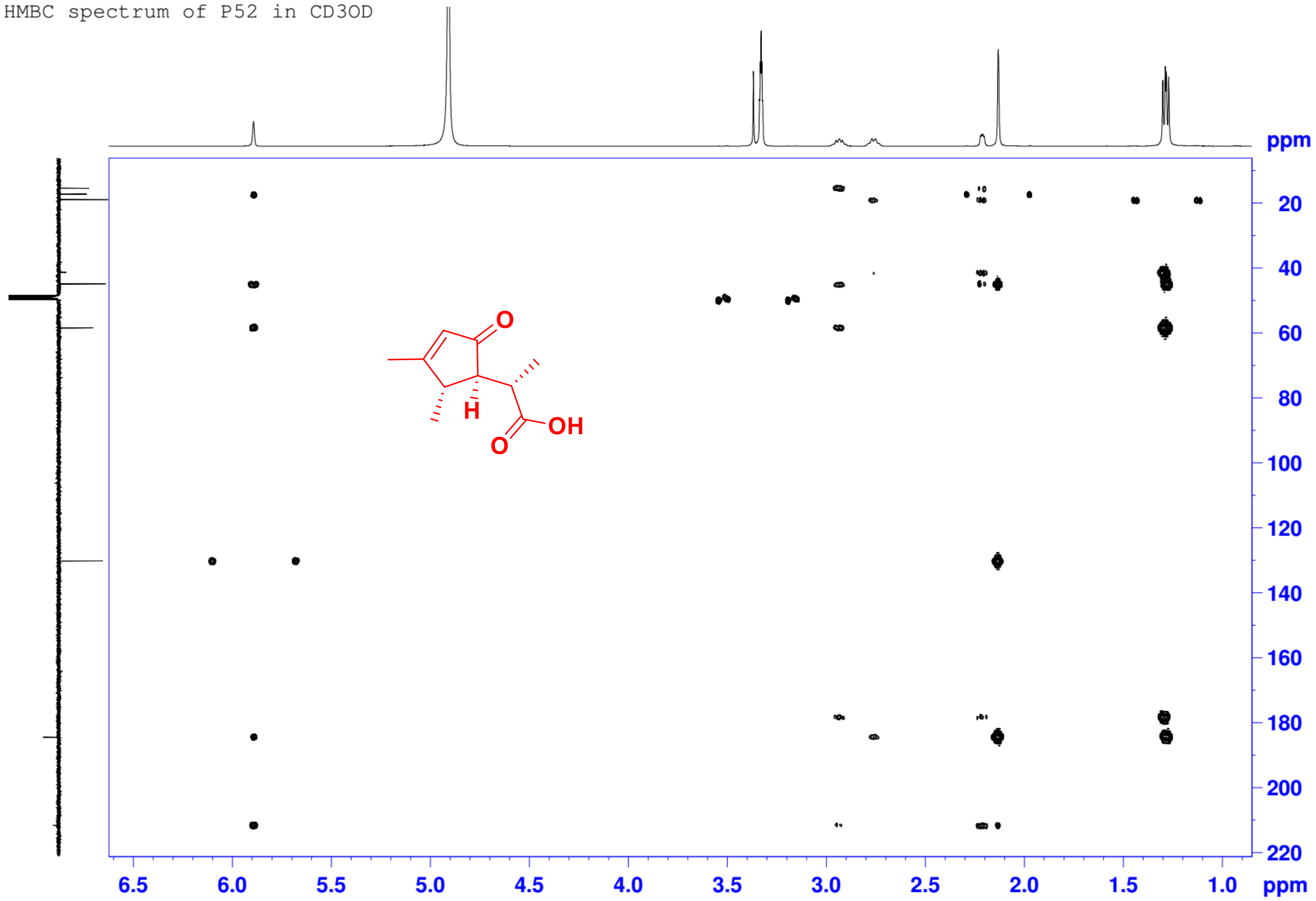

Figure S2-6. HMBC spectrum of **2** in CD_3OD.

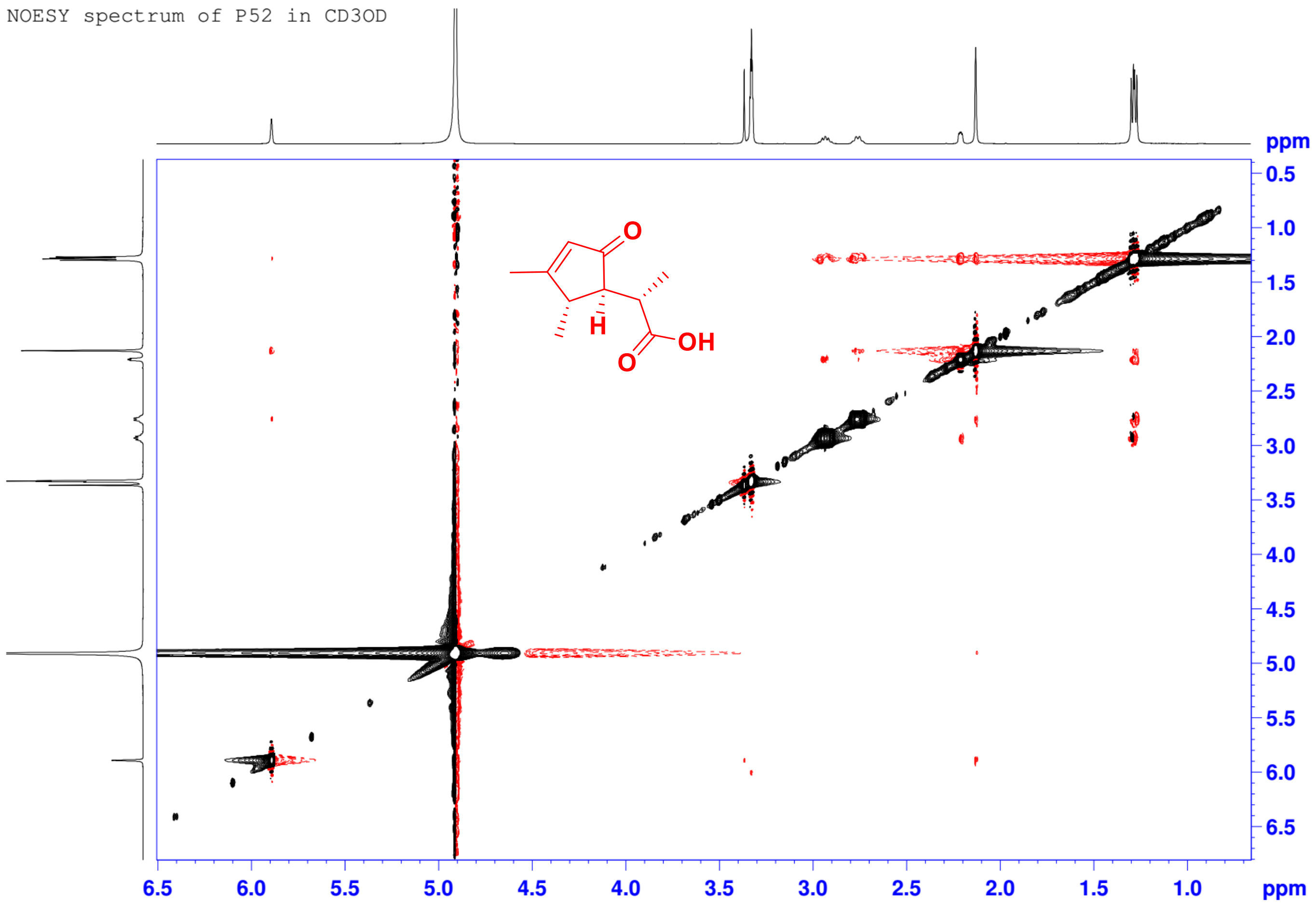

Figure S2-7. NOESY spectrum of **2** in CD_3OD.

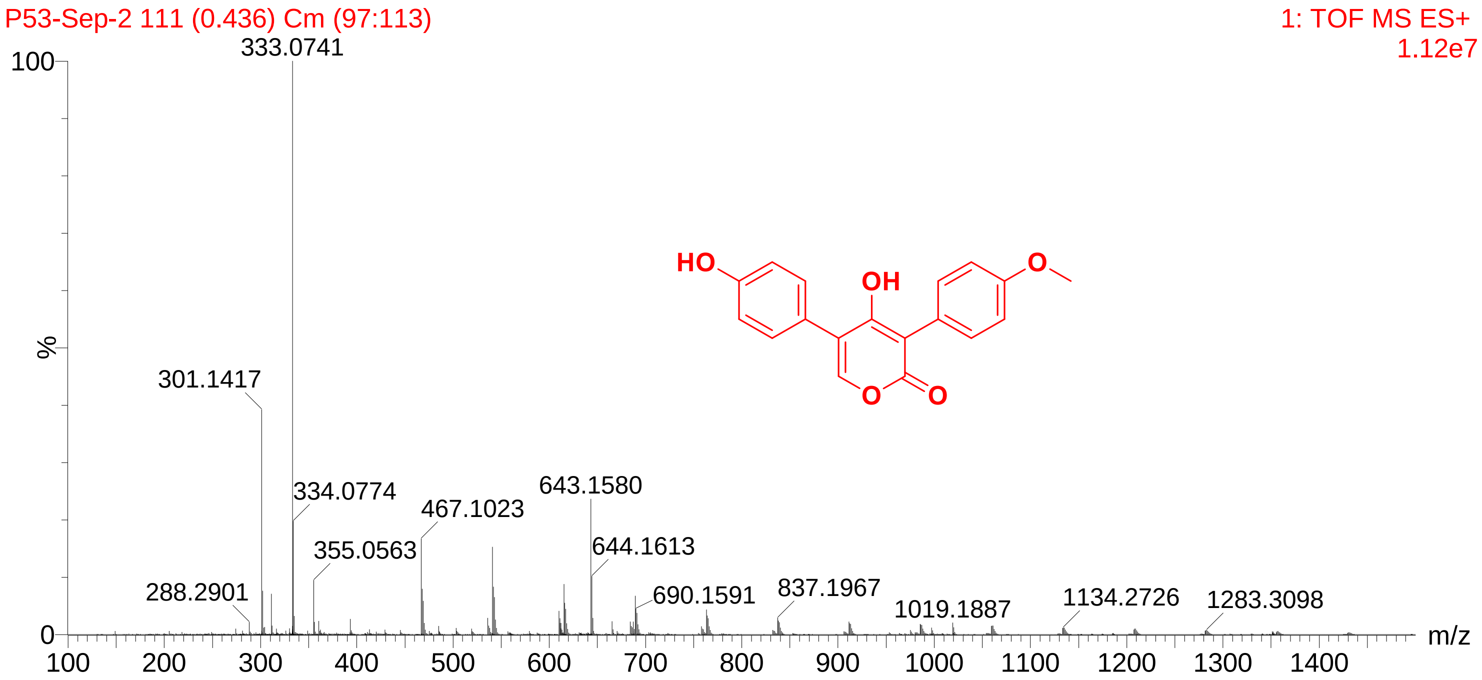

Figure S3-1. HRESIMS spectrum of **3**.

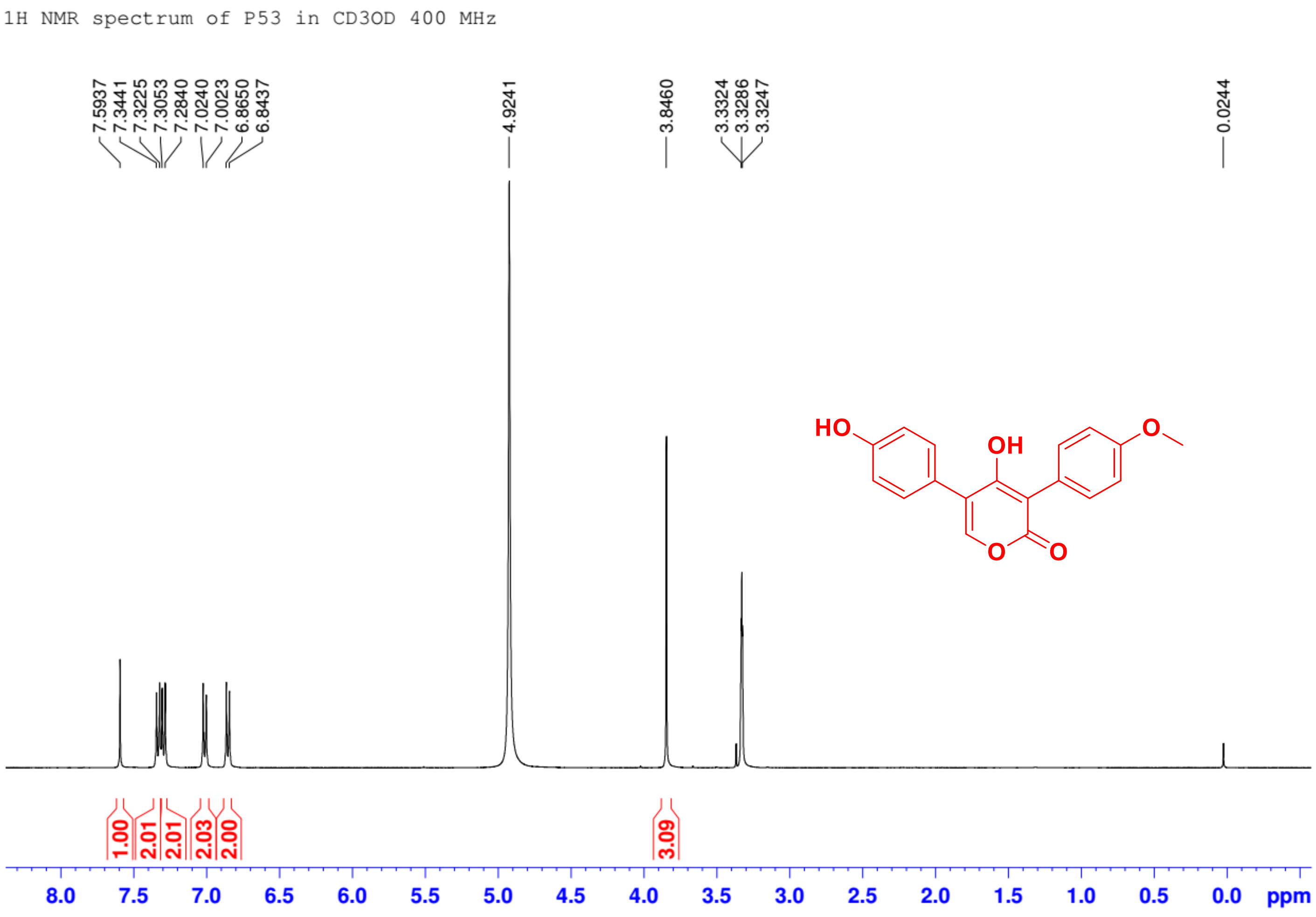

Figure S3-2. ^{1}H NMR spectrum of **3** in CD_3OD (400 MHz).

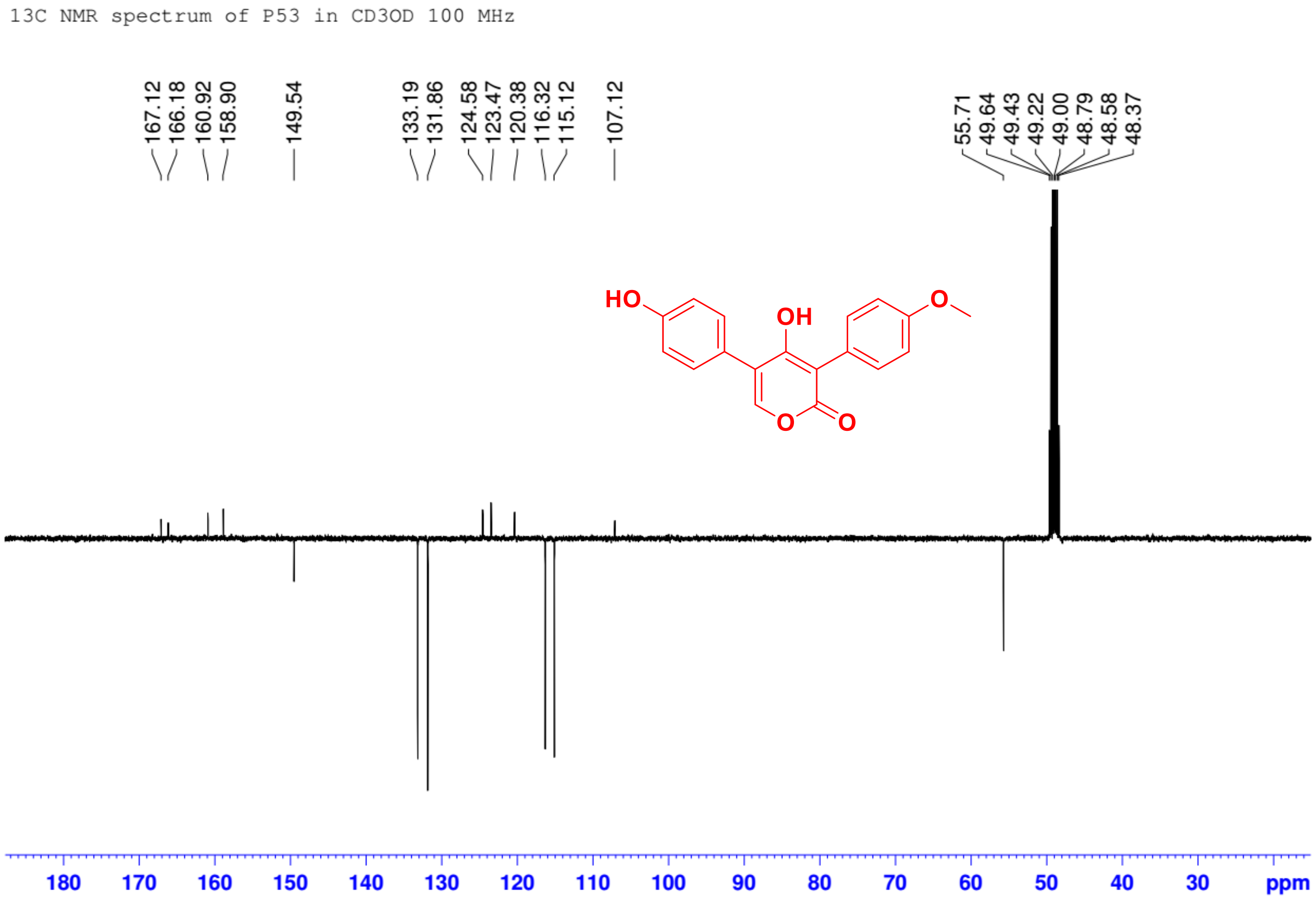

Figure S3-3. ^{13}C NMR spectrum of **3** in CD_3OD (100 MHz).

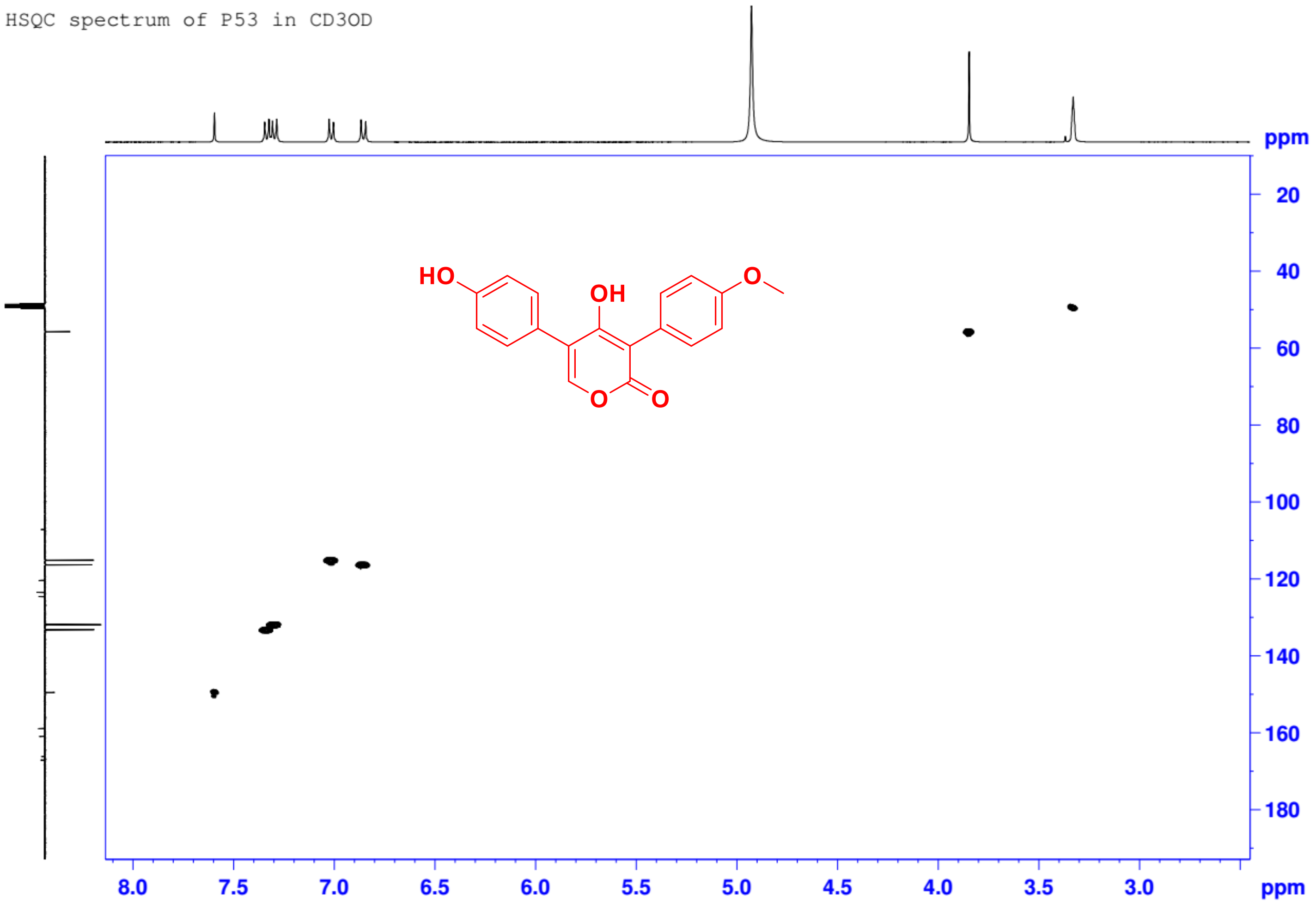

Figure S3-4. HSQC spectrum of **3** in CD_3OD.

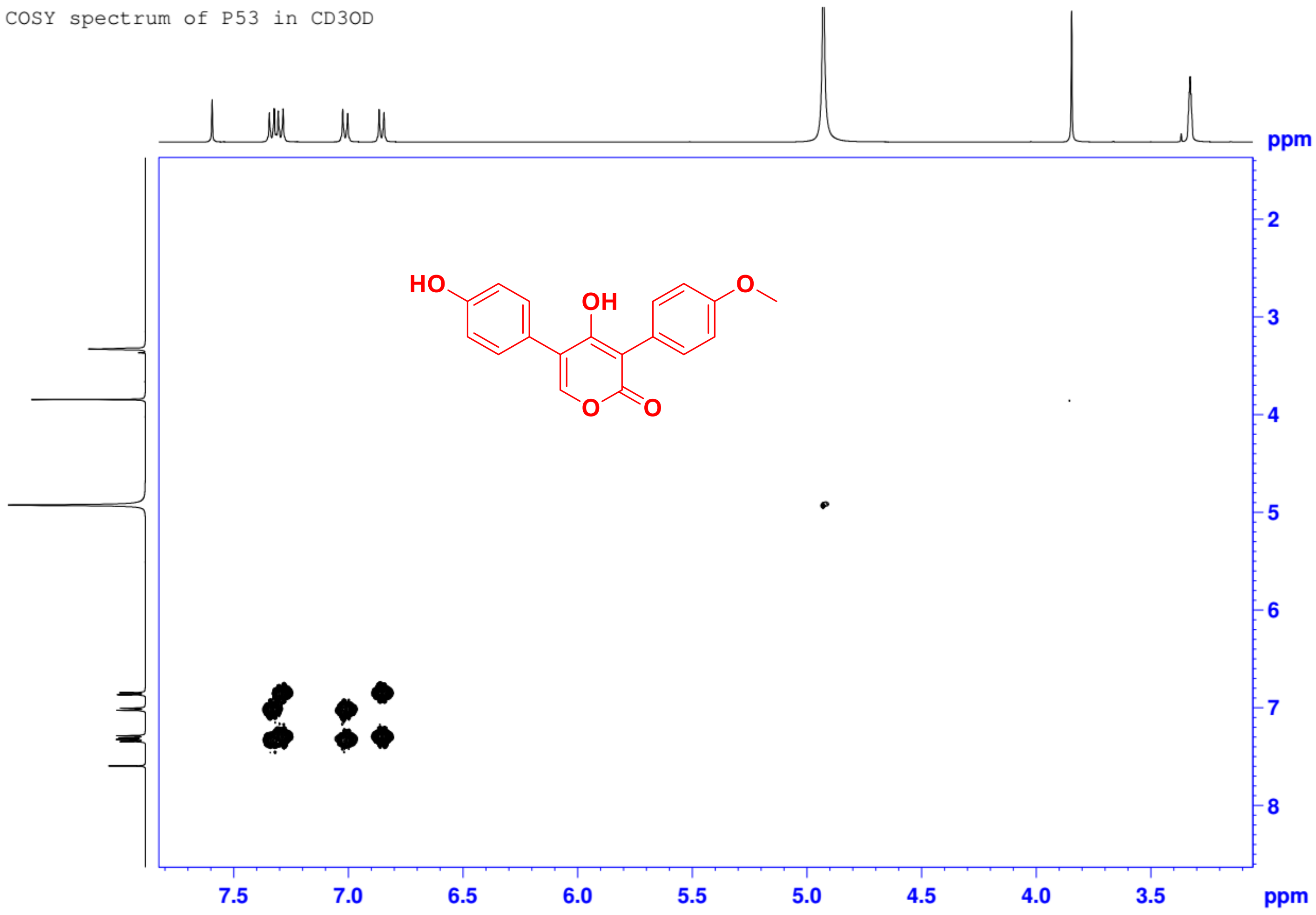

Figure S3-5. COSY spectrum of **3** in CD_3OD.

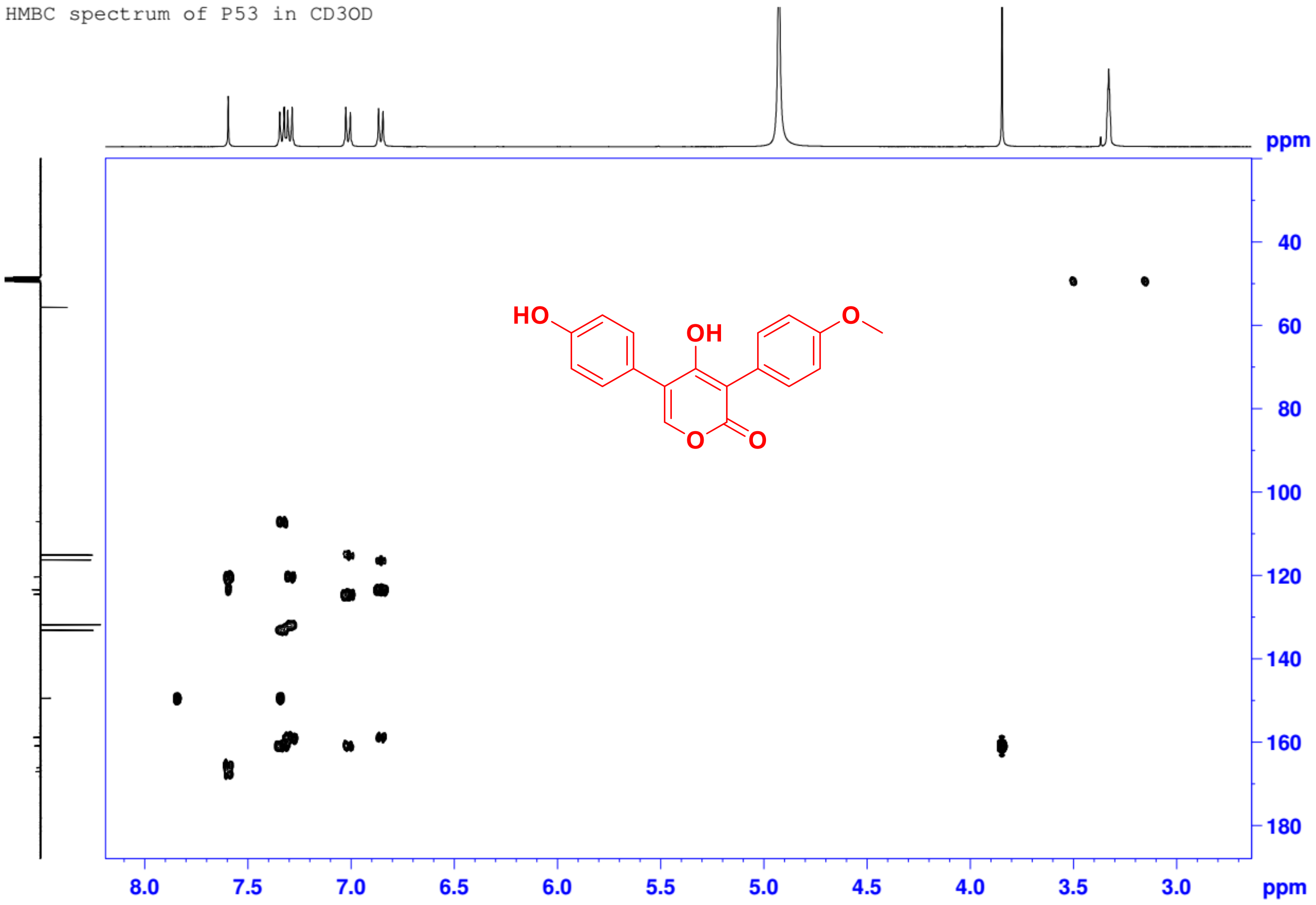

Figure S3-6. HMBC spectrum of **3** in CD_3OD.

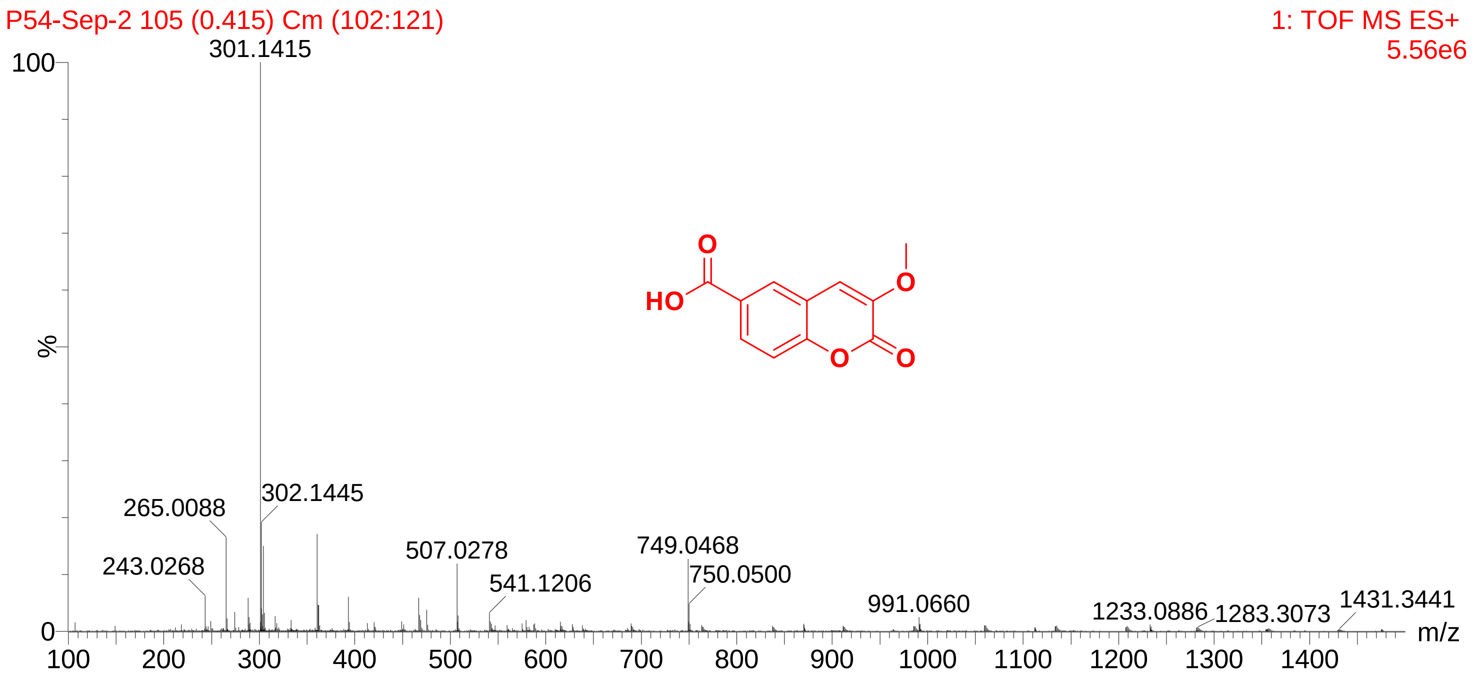

Figure S4-1. HRESIMS spectrum of **4**.

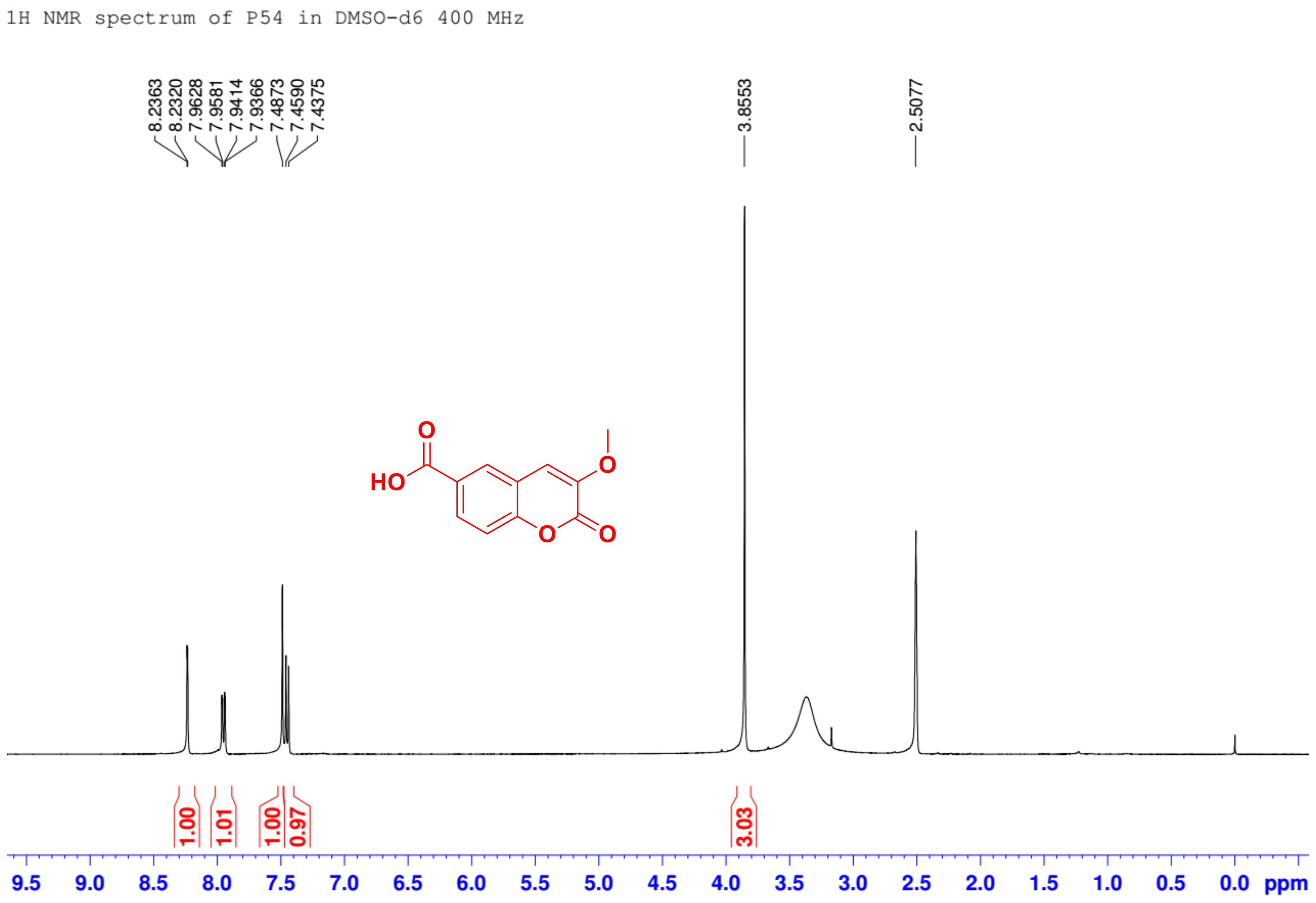

Figure S4-2. ^{1}H NMR spectrum of **4** in DMSO-d_6 (400 MHz).

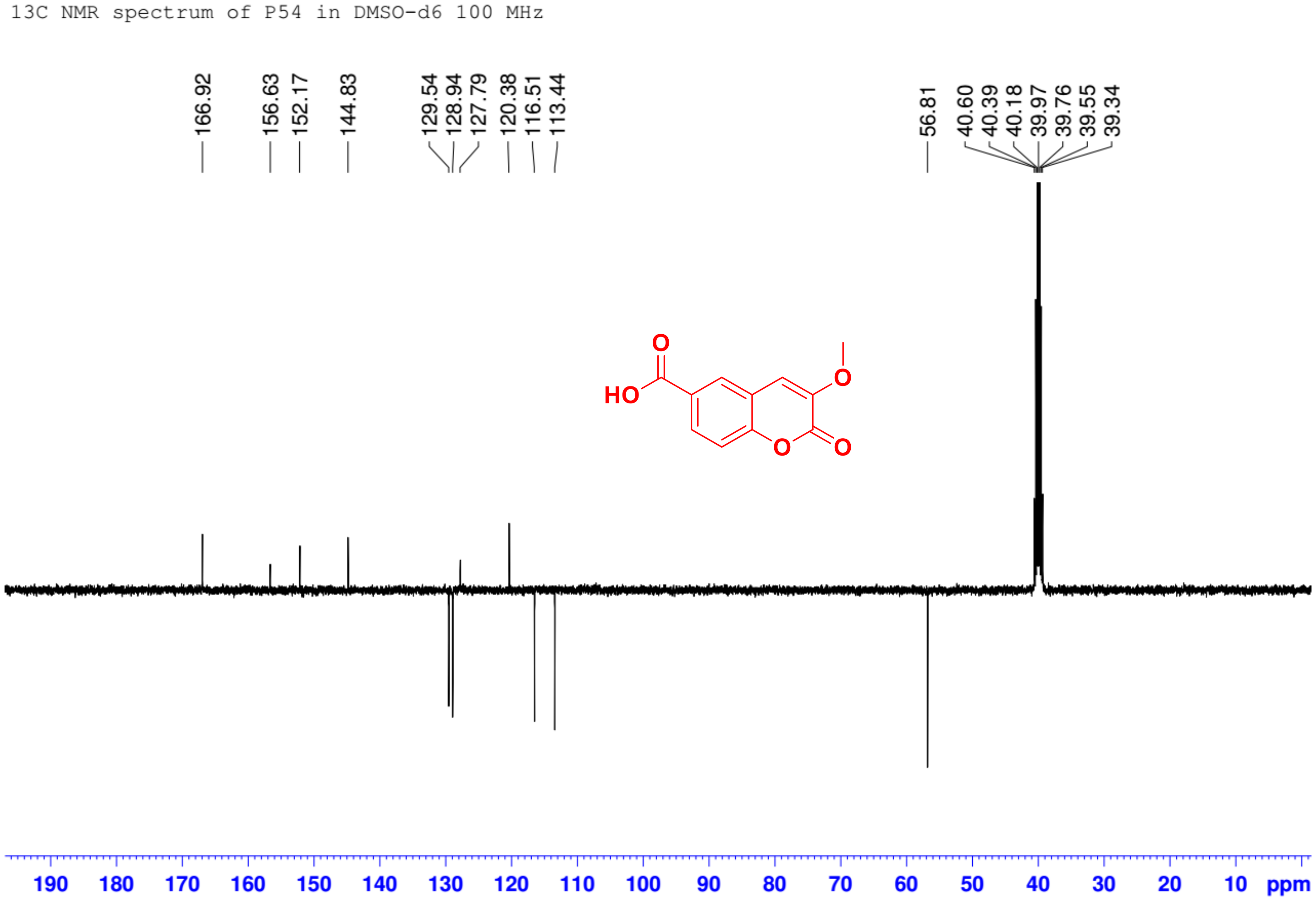

Figure S4-3. ^{13}C NMR spectrum of **4** in DMSO-d_6 (100 MHz).

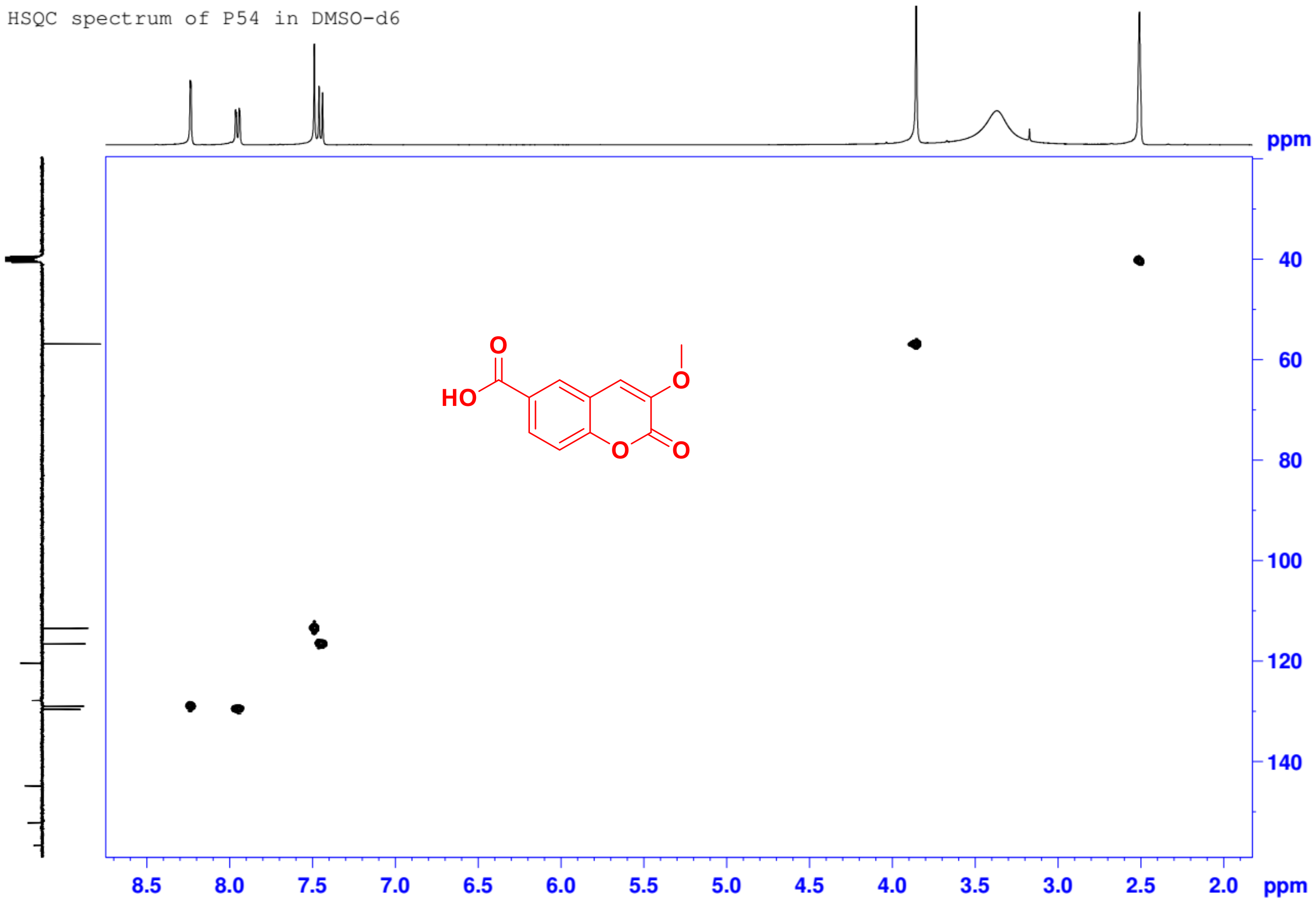

Figure S4-4. HSQC spectrum of **4** in DMSO-d_6.

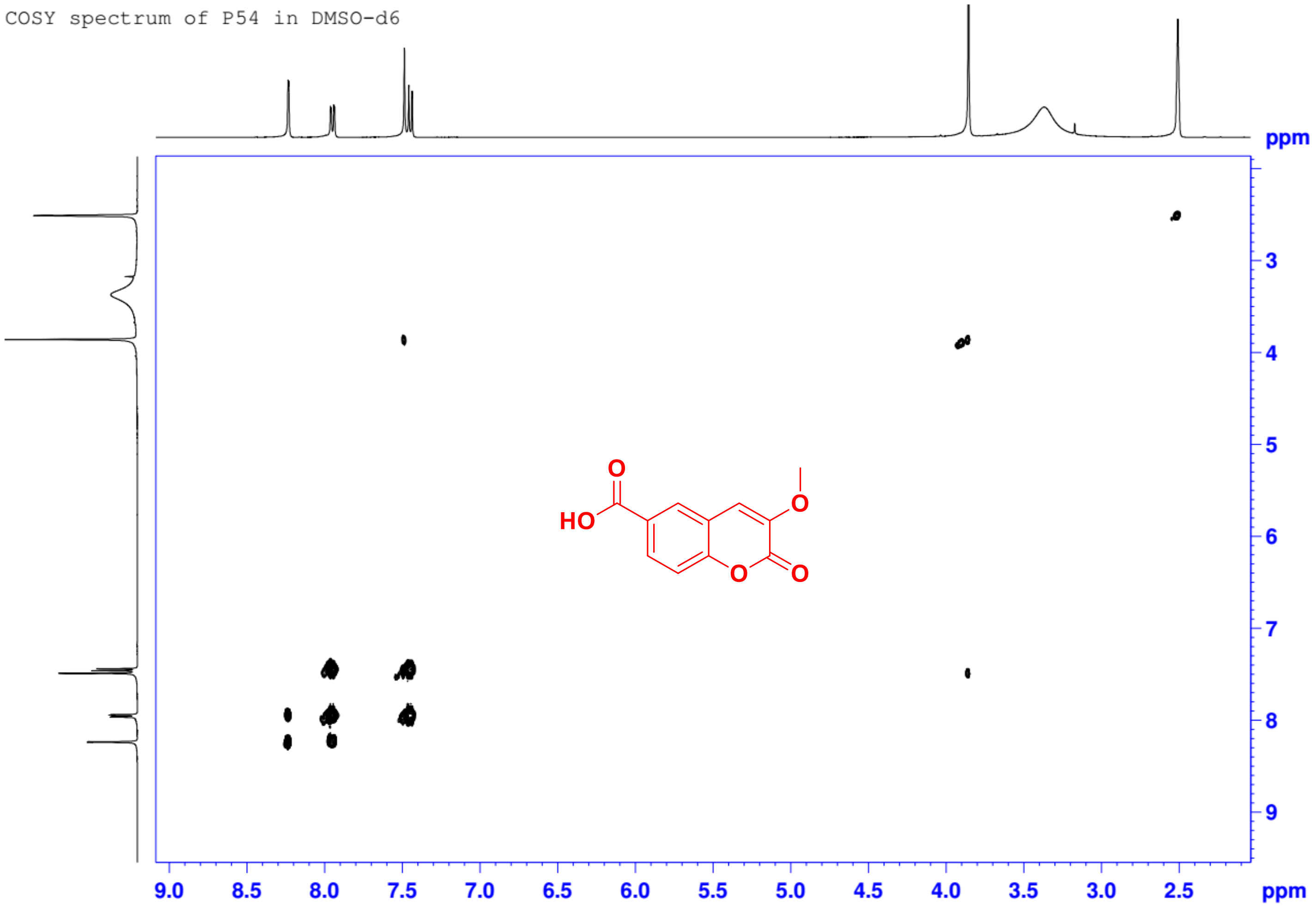

Figure S4-5. COSY spectrum of **4** in DMSO-d_6.

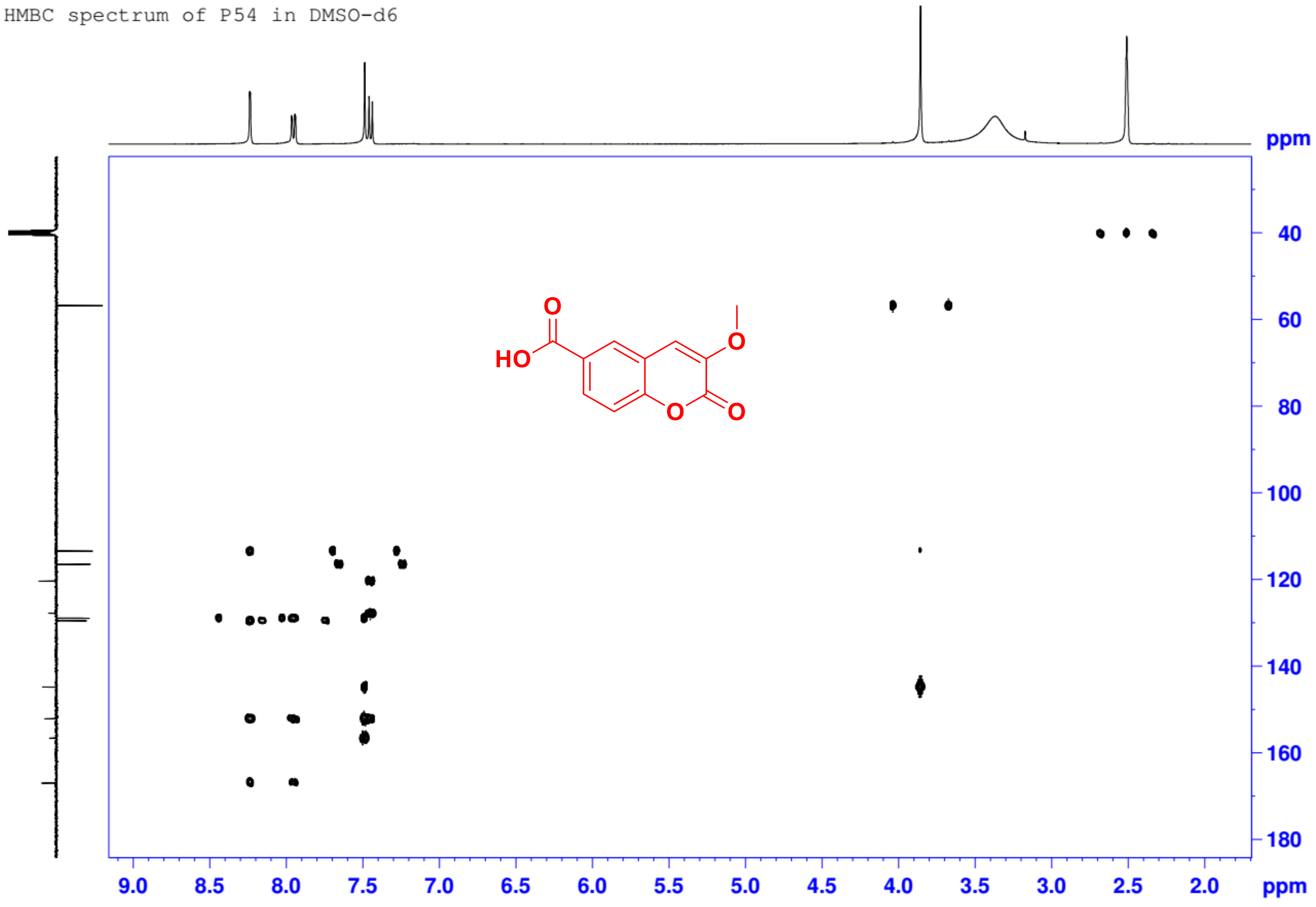

Figure S4-6. HMBC spectrum of **4** in DMSO-d_6.
